# MiR-7 inhibits progression of glioblastoma by impairing autophagy resolution, energy metabolism and ECM remodeling

**DOI:** 10.1186/s13046-025-03504-6

**Published:** 2025-08-14

**Authors:** Marta Torrecilla-Parra, Virginia Pardo-Marqués, Antonio C. Fuentes-Fayos, Miguel E. G-García, Mario Fernández-de Frutos, José L. López-Aceituno, Cristina Puigdueta, Carmen Zamora, Ana Pérez-García, Juan F. Aranda, Rebeca Busto, Manuel D. Gahete, Raúl M. Luque, Cristina M. Ramírez

**Affiliations:** 1 IMDEA Research Institute of Food & Health Sciences, Madrid, Spain; 2 Maimonides Biomedical Research Institute of Cordoba (IMIBIC), Reina Sofia University Hospital, Córdoba, Spain; 3 Department of Cell Biology, Physiology and Immunology, University of Córdoba, Córdoba, Spain; 4 Department of Genetics, Physiology and Microbiology, Faculty of Biology, Complutense, University of Madrid, Madrid, Spain; 5 Department of Biochemistry-Research, Hospital Universitario Ramón y Cajal, IRYCIS, Madrid, Spain

**Keywords:** GBM, MiR-7, Autophagy, Metabolism, ECM

## Abstract

**Background:**

Due to the poor prognosis of patients suffering malignant brain tumors such as glioblastoma multiforme (GBM), the search for new therapeutic strategies with more efficacy and higher survival rate is of utmost urgency. Growing evidence suggests that alterations in autophagy and metabolism critically contribute to the pathogenesis and progression of GBM. In this context, microRNAs are known to regulate autophagy and associated cellular functions, which point them as promising therapeutic candidates. We previously established the role of miR-7 in regulating relevant metabolic pathways related to insulin signaling and cholesterol homeostasis.

**Methods:**

Bioinformatics analysis was performed to identify miR-7 target genes potentially involved in the regulation of metabolism and cellular processes related to GBM. Ectopic expression of miR-7 was assessed to investigate its role in macroautophagy and energy metabolism. In vivo, miR-7 levels were restored in a mouse GBM xenograft model to evaluate its potential therapeutic effect in already established tumors. Additional mechanistic approaches, including transcriptomics, bioinformatics, and histopathological analyses, indicate that miR-7 modifies the tumor phenotype by altering key genes involved in extracellular matrix (ECM) remodeling in vivo.

**Results:**

Herein, we unveiled new conceptual and functional pathophysiological avenues in GBM, with potential therapeutic implications, by demonstrating a novel dual role of miR-7 on the regulation of metabolism, through the impairment of the mitochondrial function and glycolysis, and autophagy, by inducing the initiation process through the regulation of PI3K/AKT/mTORC1 signaling, while blocking later stages via posttranscriptional inhibition of two key SNARE proteins, STX17 and SNAP29. Furthermore, in vivo studies using a preclinical model showed that miR-7 overexpression in already established GBM tumors promotes a significant inhibition of tumor size and progression and replicates the metabolic defects found in vitro. Moreover, our novel findings indicate that miR-7 modifies the tumor phenotype by promoting alterations in its mechanism of extracellular matrix remodeling in vivo.

**Conclusion:**

Altogether, our study provides solid, convincing evidence demonstrating that miR-7 might be used as a promising therapeutic target for GBM, paving the way to explore its potential as novel biomarker and actionable target candidate for this lethal cancer.

**Supplementary Information:**

The online version contains supplementary material available at 10.1186/s13046-025-03504-6.

## Background

Glioblastoma multiforme (GBM) is the most aggressive malignant brain tumor, characterized by a high incidence (3–5 cases per 100,000 inhabitants), a low survival rate, and the worst prognosis among infiltrative gliomas, with no effective therapy currently available [1, 2]. Treatment typically involves tumor resection, radiotherapy, and chemotherapy with temozolomide (TMZ). However, the challenge posed by the blood-brain barrier (BBB) to drug delivery, the impossibility of achieving complete tumor resection, and treatment resistance significantly hinder clinical intervention, underscoring the urgent need for novel treatments. In this regard, strategies focused on modulating tumor metabolism have gained increasing attention, not only for their direct influence on tumor progression but also for their potential to trigger autophagy-dependent vulnerabilities in cancer cells [3]. Macroautophagy (hereafter autophagy) is a tightly regulated, multistep catabolic process involving the lysosomal degradation of cellular defective organelles and proteins, which are recycled into metabolic precursors [4]. Alterations in the autophagic process are linked to many diseases including cancer and brain tumors [5]. While the role of autophagy in cancer is complex and likely dependent on the cellular background and environmental cues, it´s widely accepted that in the tumor microenvironment, autophagy enhances cancer cell survival under metabolic stress [6]. Recently, the use of mammalian target of rapamycin complex 1 (mTORC1) and phosphatidylinositol 3-kinase (PI3K) inhibitors that promote the initiation of autophagy in combination with blockers of the late resolution phase has shown to enhance cytotoxicity and to promote autophagy-induced tumor cell death in GBM [7].Besides pharmacological intervention, microRNAs (miRNAs) have garnered interest due to their potential to regulate almost all known cellular processes, including autophagy [8], making them attractive candidates for novel therapeutic strategies in many human diseases, traditionally cancer [9]. MiRNAs are small non-coding RNAs that regulate gene expression posttranscriptionally, acting through direct binding to the 3′UTR of target mRNAs, repressing translation, inducing mRNA degradation or both [10]. Dysregulated levels and differential expression patterns miRNAs and their targets have been found during cancer and GBM [11]. Specifically, miR-7, which is highly represented in the brain, is abnormally reduced in GBM, and has been associated with the inhibition of tumor proliferation by blocking epidermal growth factor receptor (EGFR) and PI3K [12].

Previous studies from our group have demonstrated that miR-7 targets key regulators of insulin homeostatic functions, including insulin receptor (INSR) or insulin-degrading enzyme (IDE), as well as key pathways in cholesterol metabolism such as liver X receptors (LXR), pointing this miRNA as a central modulator of cellular pathways linked to human metabolic disorders [13, 14]. Building on this knowledge and in previous studies showing the specific role of miR-7 regulating GBM tumor proliferation, we set out to investigate additional metabolic actions of this miRNA on GBM and its therapeutic potential as GBM inhibitor in already established tumors. In this work, we show that miR-7 negatively affects metabolism in GBM cells by impairing mitochondrial function and glycolysis. We also reveal its novel dual role on autophagy, inducing the initiation of this critical cellular process through PI3K/AKT/mTORC1 signaling, while inhibiting later stages via posttranscriptional regulation of two *N*-ethylmaleimide-sensitive factor attachment protein receptors (SNARE) proteins, syntaxin 17 (STX17) and synaptosomal-associated protein 29 (SNAP29) as well as other lysosomal targets. Furthermore, using preclinical mouse model that allows for the restoration of miR-7 levels on demand in pre-established tumors, we were able to replicate our in vitro findings and significantly inhibit tumor size and progression. Additionally, histological and transcriptomics studies of tumor specimens revealed that miR-7 overexpression modified the tumor phenotype by altering the expression of genes involved in extracellular matrix (ECM) remodeling.

Despite the molecular link between autophagy and metabolism in cancer, and its possible regulation at posttranscriptional level, there are currently no studies addressing the impact of miR-7 on these processes in GBM. Overall, this work underscores the multifaceted therapeutic potential of miR-7, which simultaneously operates at multiple levels through posttranscriptional regulation of energy metabolism, dual modulation of autophagy in GBM, and its influence on novel processes associated with the tumor microenvironment.

## Methods

### Study design

This study investigated the oncometabolic effect of miR-7 against GBM. We used molecular and biochemical assays to unveil miR-7´s dual role in autophagy and metabolism in GBM. We also examined the impact of miR-7´s restoration in vivo in a mouse xerograph model of GBM. All experimental procedures in mice were carried out according to the European Regulations for Animal Care, under the university and regional government research ethics committees (ref 19/04/ 2023/13), approved by the Institutional Animal Care and Use Committee. Tumor samples were histological and characterized and transcriptomic analyses of tumor specimens revealed that miR-7 overexpression modified the tumor phenotype by altering the expression of genes involved in extracellular matrix (ECM) remodeling.

### Bioinformatics

Predicted miR-7 targets found in the database miRDB [15] were processed for gene ontology analysis using ShinyGO [16],PANTHER v.17.0 [17] and g: Profiler [18] to analyze cellular component enrichment and the results were represented in Cytoscape 3.9.1 (©2001–2018 Cytoscape Consortium) [19]. EncoRi [20] was used to further validate the predicted miR-7 targets in experimental datasets including CLIP-seq and to compare the miRDB-predicted targets throught additional prediction tools, including TargetScan, miRmap, miRWalk, and miRTarBase. Binding sites for miR-7 in target genes were identified using TargetScan [21, 22].

### Cell culture treatments and transfections

Mouse and human neuroblastoma cell lines (N2a and SH-SY5Y, respectively), monkey and human kidney cell lines (COS-7, HEK293, respectively), and the human glioblastoma U87-MG cell lines were obtained from the American Type Tissue Collection (ATTC, USA) and were maintained in Dulbecco’s Modified Eagle Medium (DMEM) supplemented with 10% Fetal Bovine Serum (FBS) and 1% penicillin-streptomycin in 10 cm^2^ dishes at 37 °C and 5% CO_2_. For some experiments, cells were treated with 100 µM of chloroquine for 6 h or 200 nM of rapamycin for 24 h, and for starvation experiments, cells were maintained in Hank’s Balanced Salt Solution (HBSS). For transfection experiments, cells (~ 70% confluence) were transfected with 40-60 nM mi*RIDIAN* miRNA mimic (miR-7-5p) or non-targeting control mimic sequence (CM) (Dharmacom) using Lipofectamine ^TM^ RNAimax (Invitrogen) for 48 h. In some experiments 1 µg of plasmids expressing mCherry-GFP-LC3 tandem construct, or the coding sequence (ORF) of SNAP29 or STX17 (Origene) without the 3’UTR were co-transfected with CM or miR-7-5p mimic and CM using Lipofectamine 2000 (Invitrogen).

### Generation of the U87-WT and U87-miR-7 stable cell lines

Lentiviral virus particles containing a Dharmacon™ SMARTvector™ Inducible Lentiviral shMIMIC plasmid (Horizon, GSH11929), either control (U87-Ctrl) or a miR-7-5p-doxycycline-inducible-expressing one (U87-miR-7), were generated. Briefly, HEK293 cells were transfected with the ViraPower™ Lentiviral Packaging Mix (Invitrogen, K497500) and the inducible lentiviral plasmids with Lipofectamine™ 2000 for 48 h. The medium was changed to a fresh medium every 12 h and the extracted medium was filtered and treated with polybrene at 8 µg/mL (Sigma, H9268). U87-MG cells were infected with said particles for 48 h before being selected under 1 µg/mL puromycin (Sigma, P8833) treatment. After selection, GFP-positive cells were sorted in a BD FACSAria Fusion (Benton Dickinson).

### RNA isolation and quantitative Real-Time PCR

Total RNA from cells or tissue was isolated using QIAzol (Qiagen, 50979306) according to the manufacturer’s protocol. 1 µg of total RNA was reverse transcribed to cDNA using qPCRBIO cDNA Synthesis Kit (PCRBIOSYSTEMS, K7PB30.11-10), and quantitative real-time PCR (qPCR) was performed in using qPCRBIO SyGreen Mix Lo-ROX SyGreen real-time PCR (PCRBIOSYSTEMS, 7PB20.11–05) on a QuantStudio 12 K Flex Real-Time PCR System (Applied Biosystems). mRNA levels were normalized to the levels of 18S rRNA or GAPDH (used as housekeeping genes). The primer sequences used are available upon request. For miRNA quantification, 1 µg of total RNA was reverse transcribed using QuantiTect Reverse Transcription Kit (Qiagen, 205311) and qPCR was performed using QuantiTect SYBR Green PCR Kit (Qiagen, 204143). Levels of mature miR-7 were detected using the miRCURY LNA miRNA PCR Assay (Qiagen, YP02119694), and normalized to the levels of SNORD68 as housekeeping miRNA (QuantiTect Primer Assay, 249900). For mitochondrial DNA (mtDNA), total DNA from cells was extracted using the DNeasy Blood & Tissue Kit (QIAGEN). After DNA isolation, mtDNA was amplified using primers specific for the genes of mitochondrial cytochrome c oxidase subunit 1 (mtCOX1), mitochondrial cytochrome c oxidase subunit 2 (mtCOX2), and NADH dehydrogenase 1 (mtND1), and normalized to genomic DNA by amplification of 18S or GAPDH [13].

### Western blot

Protein extraction and quantification from cells were performed as indicated before [23]. 20–30 µg of protein were analyzed by SDS-PAGE and transferred onto nitrocellulose membranes that were blocked with 5% BSA (w/v) in PBS and probed with the following antibodies from Cell Signaling [LC3B (2775), ULK1 (8054T), AKT (9272), p-AKT (9271), mTOR (2972), p-mTOR (2971), S6K (9202), p-S6K (9205), AMPK (2532), p-AMPK (2535) and CTSB (31718T), ENO2 (24330), LGALS8 (45586), PDK1 (3062)], from Abcam [p62 (ab56416), LAMP1 (ab208943), LAMP2 (ab25631) and VDAC1 (ab186321)], STX17 (NBP1-93968) from Novus, as well as RAB7 (376362) and EGFR (53274) from Santa Cruz Biotechnology. Other antibodies used are SNAP29 (ProteinTech, 12704-1-AP), GFP (Origene, TA1500), and HSP90 (BD Bioscience, 610419). Fluorescence-labeled secondary antibodies included anti-mouse (Invitrogen, A21057) and anti-rabbit (Invitrogen, A10043) and bands were visualized using the Odyssey Infrared Imaging System (LI-COR Biotechnology). Densitometry analyses of the gels were carried out using ImageJ software from the NIH (http://rsbweb.nih.gov/ij/*).* Three independent experiments performed in triplicate were analyzed.

### 3’UTR luciferase reporter assays

cDNA fragments corresponding to the 3’UTR of target genes were amplified by PCR (Phusion Hot Start II DNA Polymerase, Thermo Scientific) from genomic DNA with PmeI, XhoI or NotI linkers (New England Biolabs). The PCR product was directionally cloned into the psiCHECK2™ vector (Promega). Point mutations in the seed region of the predicted miR-7-5p sites within the 3’UTR were generatedusing the QuikChange Multi Site Directed-Mutagenesis Kit (Agilent). All constructs were confirmed by sequencing. HEK293 or COS-7 cells were plated into 12-well plates and co-transfected with 1 µg of the indicated 3’UTR luciferase reporter vector and 40 nM of the miR-7-5p mimic or CM as indicated above. Luciferase activity was measured using the Dual-Glo Luciferase Assay System (Promega). Renilla-luc activity was normalized to the corresponding Firefly-luc activity and plotted as a percentage of the control (cells co-transfected with the corresponding concentration of control mimic). At least three independent experiments were performed in triplicates.

### Cathepsin B assay

For the Cathepsin B assay, U87-MG cells were transfected with CM and miR-7-5p mimics, and 24 h later, cells were incubated in HBSS for 16 h. While cells were lysed, the medium was collected, concentrated with Amicon Ultra-0.5 centrifugal filter units (Merck) and analyzed by Western blot.

### Cell staining and imaging

Cells were transfected as indicated beforeand treated with 50 µM of Monodansylcadaverine (MDC, Sigma, D4008) for 10 min to stain acidic vesicles. In the autophagy rescue experiments, cells transfected as indicated before were fixed with 4% of paraformaldehyde (PFA), incubated with blocking buffer (5% BSA, 0.3% Triton X-100 in PBS) and probedforanti-SNAP29 (ProteinTech, 12704-1-AP), or anti-STX17 (Novus, NBP1-93968) antibodies in combination with an appropriate Alexa-594 or Alexa-680-conjugated secondary antibodies, and VECTASHIELD^®^ Antifade Mounting Medium with DAPI (Vector Labs, H-1200). Specificity of the positive immunofluorescent staining was corroborated by substituting the primary antibody with a matched immunoglobulin. All preparations were analyzed using a Zeiss LSM800 laser scanning confocal microscope (Oberkochen, Germany) or a Nikon ECLIPSE Ti-epifluorescence inverted microscope (Melville, USA). All gains for the acquisition of comparable images were maintained at a constant level. Images were processed using Image J software (NIH).

### Mito stress test and Glycolysis stress test

The oxygen consumption rate (OCR) and extracellular acidification rate (ECAR) were measured by using the Seahorse XFe96 Analyzer (Agilent). Previously miR-7 or CM transfected cells were seeded at 3–4 × 10^4^ cells/well for OCR and ECAR Assays into Seahorse XF 96-culture plates (Seahorse Bioscience, 102601-100) and cultured in complete DMEM for 24 h in a 5% CO_2_ incubator at 37 °C. Then, cells were cultured in XF base medium (Seahorse Bioscience) at 37 °C for 1 h in a CO_2_-free incubator. After measuring the basal mitochondrial respiration, oligomycin (2 µM), Fluoro Carbonyl Cyanide Phenylhydrazone (FCCP; 0.3 µM) and a mixture of rotenone and antimycin A (0.5 µM) were sequentially injected into each well. OCR was recorded as pmoles per minute and calculated as a percentage of the OCR value before the treatment of tested agents. ECAR was measured after the sequential injection of glucose (10 mM), oligomycin (1.5 µM) and 2-deoxy-glucose (2-DG, 50 mM) at the specified time points, as the basal ECAR, maximal ECAR and reserve capacity. Averages of five wells were taken per data point. After the assays, plates were saved and protein concentrations for each well were measured to normalize the results. Three independent experiments of each assay were performed.

### MitoTracker, mitosox and lysotracker stainings

Cells previously transfected with miR-7 or CM were incubated with 250 nM MitoTracker™ Green FM or MitoTracker™ Red CMXRos (Invitrogen), or with 2µM MitoSOX™ Red, or 100nM LysoTracker™ Green DND-26 for 30 min at 37 °C in DMEM. After incubation, cells were washed and analyzed by flow cytometry in a BD FACSCelesta SORP (Becton Dickinson). Three independent experiments were performed in triplicates.

### Lysosomal pH measurements

Lysosomal pH in U87-MG cells was measured as previously described [24] using LysoSensor Yellow/Blue DND-160 (Invitrogen, L7545). Cells previously transfected with miR-7 or CM were seeded in black 96-well plates for fluorescence analysis or on coverslips for confocal imaging. They were incubated for 3 min at room temperature with 2 µM dye in isotonic buffer (105 mM NaCl, 5 mM KCl, 6 mM HEPES acid, 4 mM HEPES-Na, 5 mM NaHCO₃, 60 mM mannitol, 5 mM glucose, 0.5 mM MgCl₂, 1.3 mM CaCl₂, pH 7.4). After staining, cells were rinsed three times with fresh buffer. The coverslips were mounted and then imaged immediately using a Zeiss LSM800 laser scanning confocal microscope (Oberkochen, Germany). The excitation wavelength was set to ~ 405 nm and images were captured under both emission wavelengths, 450 ± 20 nm and 510 ± 20 nm. Quantification of fluorescence were performed in black 96-well plates using a CLARIOstar Plus plate reader (BMG LABTECH). Excitation was set at 340 nm and 380 nm, and emission was recorded at 527 nm. The ratio of fluorescence intensity at 340 nm to that at 380 nm was used to estimate the relative lysosomal pH. For calibration of absolute lysosomal pH, cells stained with the probe were incubated at room temperature for 10 min in calibration buffers with defined pH values (4.0, 4.5, 5.0, 5.5, 6.0, and 6.5) consisted of MES solution (20 mM 2-(N-morpholino) ethanesulfonic acid, 110 mM KCl, 20 mM NaCl) supplemented with 10 µM monensin and 30 µM nigericin to equilibrate intracellular and extracellular pH. The fluorescence ratios obtained under these conditions were plotted against the corresponding pH values to construct a standard curve for pH determination.

### Cell migration and invasion

Cell migration and invasion were evaluated as described previously [25] using Millicell Cell Culture Inserts (Sigma Aldrich, PI8P01250) following the manufacturer’s instructions, Briefly, previously transfected cells with miR-7 or CM (50,000 cells) were resuspended in 300 µL serum-free DMEM and seeded into the inserts. For invasion assays, inserts were pre-treated with 200 µL collagen type IV (Sigma-Aldrich, C5533) at 0.25 mg/mL. Inserts were placed in 24-well plates containing 300 µL complete medium (10% FBS) and incubated at 37 °C for 24 h. Migrated or invaded cells were fixed and stained with crystal violet (0.05% crystal violet, 6% glutaraldehyde). The dye was eluted with 10% acetic acid, and absorbance at 560 nm was measured. A negative control with serum-free medium in the lower chamber was included. Migration and invasion were expressed as the percentage relative to cells transfected with CM, which were considered as 100%. Three independent experiments were performed in triplicates.

### Transmission electron microscopy (TEM) analysis

Cells were transfected with miR-7 or CM, washed twice with PBS 1X and fixed in Glutaraldehyde (2%) plus PFA (4%) for 1 h at RT. Samples were post-fixed in 1% Osmium Tetroxide for 1 h at 25 °C, dehydrated in acetone and embedded in epoxy resin. Ultrathin sections were stained with Uranyl Acetate and Lead Hydroxide and observed under a transmission electron microscope at the Molecular Biology Center (CBMSO) Electron Microscopy core facility.

### Tumorspheres formation and assays

U87-Ctrl and U87-miR-7 GBM cell lines (100 cells/well) cultured in a Nunclon™ Sphera™ ultra-low attachment plate (Thermofisher, 174931) using DMEM F-12 (Gibco, 11320033) with human recombinant EGF (20 ng/mL; Thermofisher, A42556) and doxycycline (1 µg/mL) for 10 days [26]. At the end of the experiments, photographs were taken to visualize and measure the area of the tumorspheres. Proliferation was analyzed by measuring cell cycle with propidium iodide (PI, Sigma, P4864) staining by flow cytometry in a BD FACSCelesta SORP (Becton Dickinson). Briefly, tumorspheres were disaggregated and centrifuged at 300 g for 5 min and the resulting pellet was washed in PBS 1X plus 2% FBS (staining buffer) before being resuspended in Annexin V binding buffer (Bionova, NB-5000-L050). Cells were then added to a solution of ethanol and saved at -20 °C at least overnight. After washing the ethanol with the staining buffer, cells were treated with RNAse 100 µg/mL and stained with PI at 200 µg/mL at room temperature in the dark for 30 min and then analyzed in the cytometer. Three independent experiments were performed in triplicates.

### GBM xenograft model

Formation of tumors in 5-week-old ATHYM-Foxn1^nu/nu^ mice (10 mice total; Janvier Labs) was performed by subcutaneous injection of 3 × 10^6^ U87-Ctrl cells in one flank of each animal and 3 × 10^6^ U87-miR-7 cells in the other flank using 100 µL of basement membrane extract (Trevigen, 3432-010-01) per flank. Cells were allowed to proliferate until the tumor was clearly visible, measured with a digital caliper, and the calculated measured volume was 100 mm^3^. The maximal tumor burden permitted by our ethics committee was 1.5 cm in diameter or 10% of body weight, and all specimens were below this restriction. At that point, 2 g/L of doxycycline with 5% sucrose was added to the drinking water and tumors were measured every 3 days. After 20 days, mice were sacrificed and tumors were photographed, dissected, weighted, preserved in 4% PFA or liquid nitrogen, and then processed for further analyses. This study was approved by the university/regional government research ethics committees (ref 19/04/2023/13) and was conducted following the European Regulations for Animal Care.

### Histological analysis and immunostaining

Tumor sections preserved in 4% PFA were transferred to a solution of 30% sucrose for 24 h before being included in OCT, frozen solid, cut in a cryostat in sections of 4 μm and prepared for Haematoxylin-Eosin (H&E) staining at the CNB Histology Facility (Madrid, Spain). For immunofluorescence staining, frozen tumor sections were fixed in 4% paraformaldehyde for 15 min and then blocked for 1 h at room temperature with blocking buffer (5% NGS, 0.5% BSA, 0.3% Triton X-100 in PBS). Sections were incubated overnight at 4 °C with primary antibodies against LAMP1 (Abcam, ab25631), LC3 (Cell Signaling, 2775). After washing, samples were incubated with Alexa Fluor-conjugated secondary antibodies for 1 h at room temperature. Stained sections were imaged using a Nikon ECLIPSE Ti-epifluorescence inverted microscope (Melville, USA), and images were acquired under identical exposure time, gain, and offset settings. Ki-67 staining to determine proliferation status and Masson’s Trichrome staining of collagen were performed by an expert pathologist (Córdoba, Spain). Antigen retrieval and inactivation of endogenous peroxidase were performed in tumor slides before the incubation with an anti-Ki67 antibody D3B5 (Cell Signaling, 12202) followed by the ABC-HRP Kit (Vector Labs, PK-6100). Nuclei were counterstained with hematoxylin. Finally, the slides were dehydrated, cleared, and mounted for microscopic evaluation. The H-Score was calculated by dividing the number of Ki-67 positive tumor cells by the total number of counted tumor cells.

### Transcriptomic arrays

RNA from GBM tumors (1 µg) was subjected to a cDNA microarray analysis on the Clariom™ S Array (Thermofisher, 902926). Data was acquired on the GeneChip™ 3000 instrument (Affymetrix, Santa Clara, California, USA) and high-throughput automated processing was performed using the GeneTitan™ Microarray System (Thermofisher) as shown before [14]. Genes were considered significantly induced or repressed with an FDR (adjusted p-value) ≤ 0.05 and a relative change of expression ≥ 2 or ≤ 0.5 and used for posterior analyses. Custom matrisome qPCR dynamic array based on microfluidic technology (Fluidigm, BMK-M-48.48) was used to determine the simultaneous expression of 48 transcripts in 8 samples (*n* = 3 Ctrl, *n* = 3 miR-7) using the Biomark System and the Fluidigm^®^ Real-Time PCR Analysis Software v.3.0.2 and Data Collection Software v.3.1.2 (Fluidigm). Normalization was performed using three housekeeping genes [β-actin (ACTB), hypoxanthine phosphoribosyl-transferase (HPRT), and glyceraldehyde 3-phosphate dehydrogenase (GAPDH)] and the GeNorm v.3.3 software as previously reported [27]. MetaboAnalyst Software v.4.0 (McGill University, Canada) was used for the analysis of the matrisome array following the criteria indicated above.

### Statistical analysis

Statistical analysis was performed in Prism software version 6.0 or posterior (GraphPad Software, La Jolla, CA, USA). Statistical differences were assessed by t-test or one-way ANOVA followed by Bonferroni’s correction test. Values of *P* < 0.05 were considered statistically significant. Data represent means ± standard error of the mean (SEM). * *P* < 0.05; **P < 0.01; *** *P* < 0.001; **** *P* < 0.001, significantly different from control conditions.

## Results

### Bioinformatic analysis uncovers novel predicted metabolic functions of miR-7

**Fig. 1:**
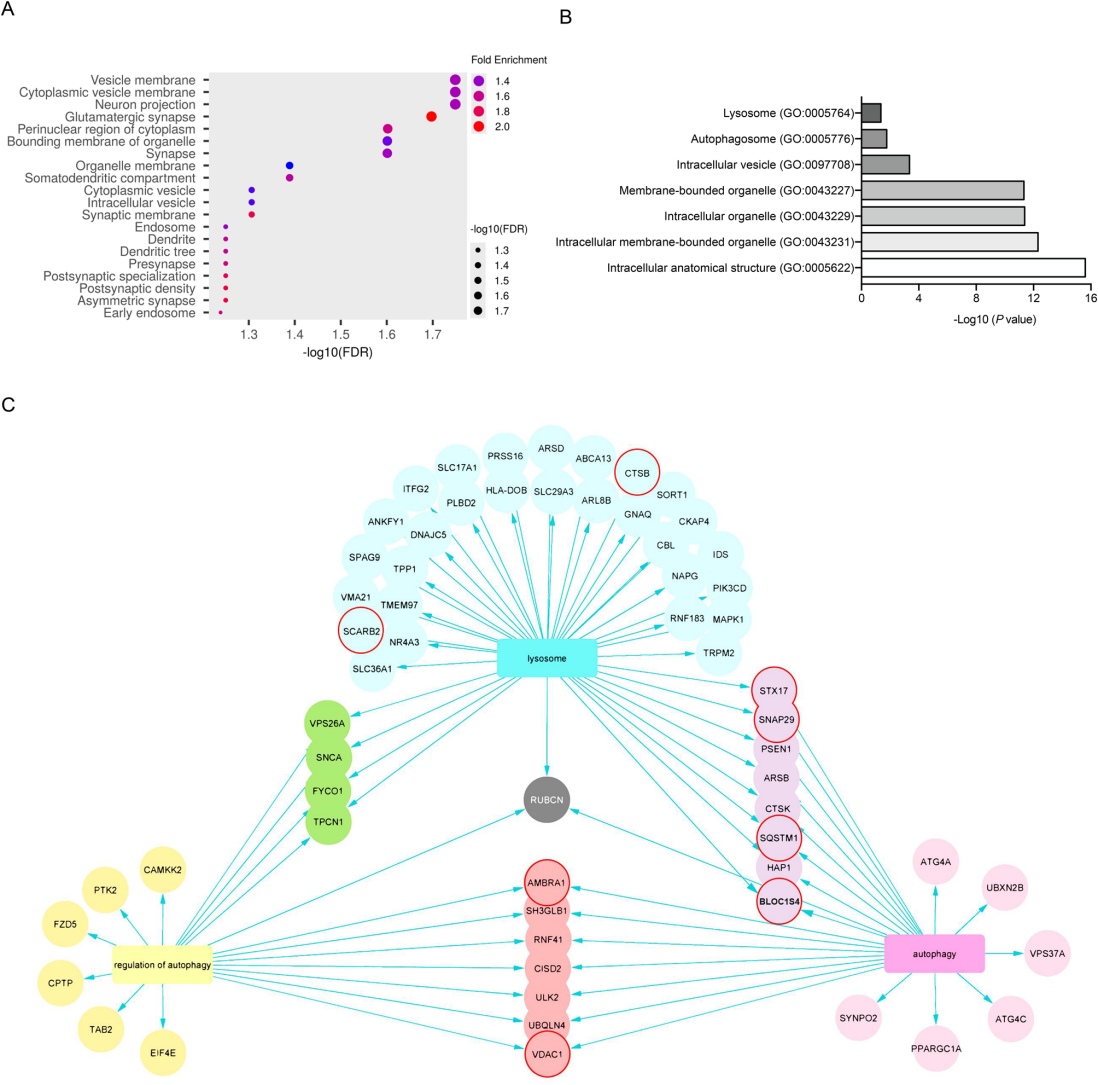
Bioinformatic analysis of predicted miR-7 targets shows an enrichment in autophagy-related proteins. **A**. Gene ontology (GO) enrichment analysis of hsa-miR-7 target genes performed using ShinyGO, highlighting significant cellular components involved. **B**. Representation of Panther GO analysis of hsa-miR-7 predicted target genes. **C**. Graphic Schematic representation of autophagy-related proteins, including the ones specific with lysosome biology, that are predicted to be miR-7 targets by miRDB. Targets that share GO Terms between 2 categories are represented in light red (autophagy-regulation of autophagy), purple (autophagy-lysosome), green (lysosome-regulation of autophagy) and gray (autophagy-regulation of autophagy-lysosome). Selected targets are circled in red

MiR-7 is an abundant miRNA in the brain, and previous studies by our group showed that it regulates relevant metabolic functions, such as insulin signaling and cholesterol synthesis [13, 14, 28]. Although still by an unknown mechanism, miR-7 is abnormally downregulated in GBM, which is associated with tumor progression, likely due to its antiproliferative actions through the posttranscriptional inhibition of the PI3K and EGFR pathways [11]. However, the role of miR-7’s metabolic effects in GBM and their therapeutic potential is not yet fully elucidated. To explore this, we implemented a bioinformatic analysis using miRDB to obtain predicted hsa-miR-7 target genes [15] and perform gene ontology (GO) analysis of cellular components using ShinyGO and Panther [16, 17]. We observed a significant enrichment of genes related to neuronal components such as dendrites and synaptic membranes, as expected given the relevance of this miRNA in the brain, together with a notable enrichment in genes involved in vesicle formation (Fig. 1A). A deeper analysis using complementary methods available in the Panther database confirmed these findings, showing a significant increase in targets related to intracellular vesicles, autophagosomes, and lysosomes (Fig. 1B). The identified miR-7 targets belonging to these cathegories including as BLOC1S4 (Biogenesis of Lysosome-related organelles Complex 1 Subunit 4), CTSB (Cathepsin B), STX17 (Syntaxin 17), SNAP29 (Synaptosomal-associated protein 29), or LimpII-SCARB2 (Lysosome membrane protein 2-Scavenger receptor class B member 2), were represented, together whith their interconnections in Fig. 1C, Suppl Fig. 1A. Additionally, to enhance the robustness of our findings, we conducted an EncoRi analysis displaying the number of CLIP-seq experiments supporting miR-7 binding to the selected targets (Suppl Fig. 2A). We also cross-validated these targets against additional prediction tools, including TargetScan, miRmap, miRWalk, and miRTarBase (Suppl Fig. 2A). Furthermore, we performed an independent Gene Ontology analysis using g: Profiler as an additional validation platform, which consistently revealed enrichment of cellular components such as lysosomal membranes and autophagosomes, in line with the results obtained with other tools (Suppl Fig. 2B).

### MiR-7 promotes accumulation of autophagic-like vesicles and autophagy markers

**Fig. 2:**
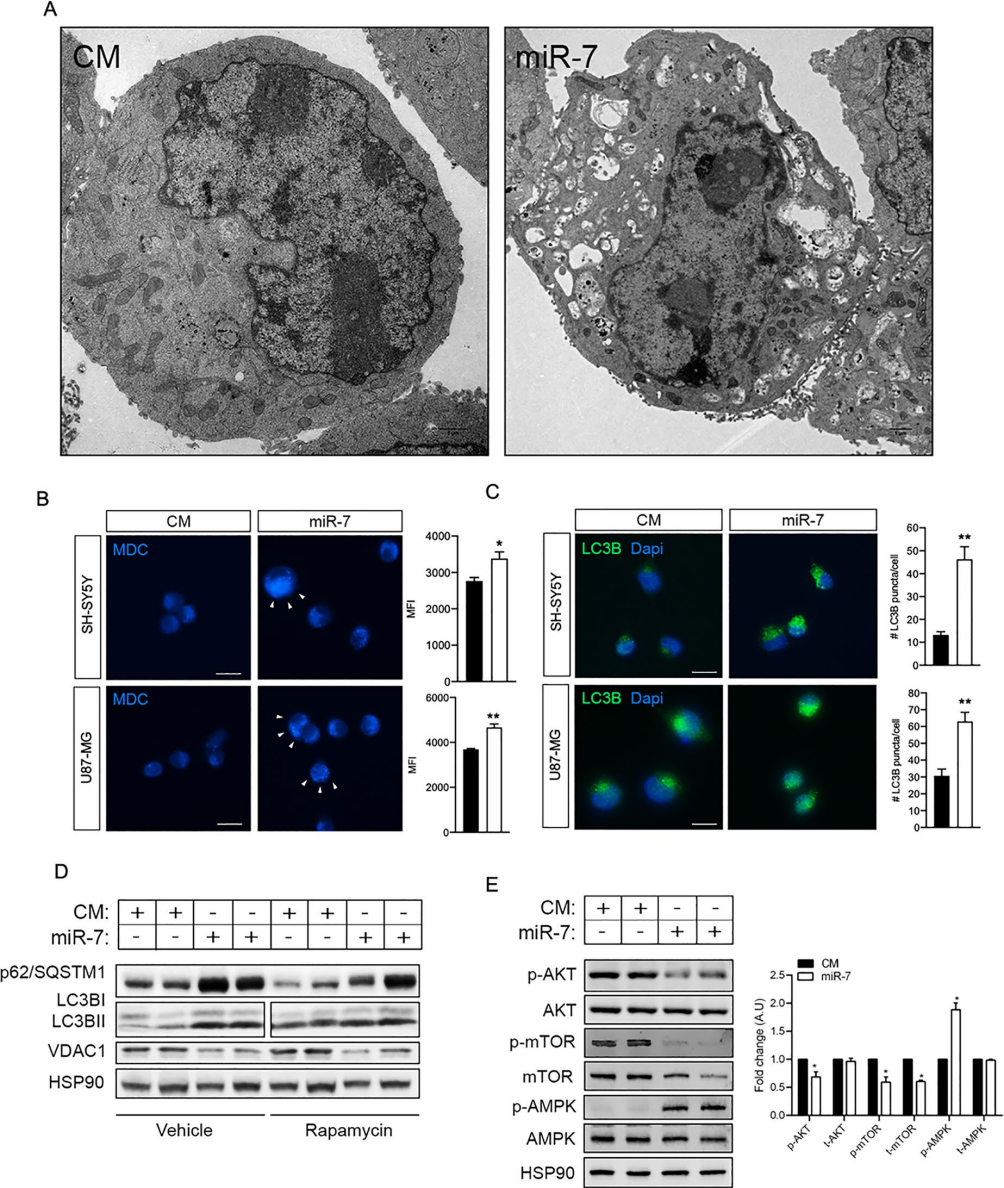
MiR-7 produces an accumulation of autophagy vesicles in vitro. **A**. Representative electron microscopy images showing the general morphology and intracellular contents of U87-MG cells transfected with control mimic (CM) or miR-7-5p mimics (miR-7). Scale bar: 1 μm. **B**. Representative confocal microscopy images of monodansylcadaverine (MDC) staining in SH-SY5Y and U87-MG transfected with CM or miR-7. Scale bar: 10 μm (Right) Mean fluorescence intensity of MDC measured by flow cytometry. Data represent means ± standard error of the mean (SEM). *P < 0.05; **P < 0.01, significantly different from CM conditions. **C.** Confocal immunofluorescence imaging of LC3B (green) in cells treated as in A, B). Dapi (blue) was used to stain the nucleus. Quantification is shown in the right and represents the number of LC3B puncta per cell (*n* = 3 cells per field, 6 fields per experiment). Data represent the mean of three individual experiments ± SEM (**P < 0.01 compared with CM). Scale bar: 10 μm. **D**. Representative Western Blots of autophagy markers p62/SQSTM1 and LC3BI/II in U87-MG cells transfected with CM or miR-7 and treated with rapamycin (200 nM) or vehicle for 24 h. VDAC1 was used as positive control of miR-7 transfection efficacy and HSP90 as loading control. **E**. Representative Western Blot of total AKT, mTOR and AMPK proteins and their phosphorylation forms in U87-MG cells transfected with CM or miR-7. Right panels show relative protein expression normalized to that of HSP90. Data correspond to the means ± SEM of three experiments performed in triplicate. *P < 0.05, significantly different from cells transfected with CM (normalized to 1)

To study the relevance and validate the findings obtained in the bioinformatic analysis, we transfected mouse and human neuronal cells and human glioblastoma U87-MG cells with miR-7 or a control mimic (CM) and processed them for electron microscopy (EM) imaging. Ultrastructural analysis showed a significant accumulation of vesicles with characteristic features of autophagosomes such as a double membrane and electron-dense, organelle-like content in miR-7 overexpressing U87-MG cells, compared to control cells (Fig. 2A), that was later corroborated by the MDC staining, which specifically marks autophagic vacuoles (Fig. 2B). We further explored the accumulation of autophagic vesicles by analyzing two well-known autophagy markers microtubule-associated protein 1 A/1B-light chain 3 (LC3B) and sequestosome 1 (SQSMT1/p62) in the presence of miR-7 and rapamycin. Our results showed that miR-7 overexpression led to a significant increase of LC3BII by Western blot (Fig. 2D), and LC3B-positive punctuate by immunofluorescence (Fig. 2C), effect that was accompanied by a substantial accumulation of p62 (Fig. 2D). As expected, treatment with rapamycin promoted the activation of autophagy and reduced p62 expression in control transfected cells, while this effect was blunted in the presence of miR-7, wherein p62 protein persisted at elevated levels (Fig. 2D). The simultaneous accumulation of LC3B and p62, together with the significant increase of intracellular autophagosome-like vesicles suggested an impairment of the autophagy process induced by miR-7. However, despite this accumulation of autophagic markers, the analysis of the classical signaling pathways controlling the initiation of autophagy indicated that miR-7 led to a downregulation of autophagy inhibitors mTOR and AKT, together with the increased phosphorylation and activation of the autophagy inducer AMPK (Fig. 2E). Indeed, mTOR, the main protein in the autophagy-inhibitory complex mTORC1 was not only less activated upon miR-7 overexpression, as seen by its reduced phosphorylated state, but also less expressed, which correlates with the results obtained by qPCRs (Supp Fig. 1B, C). The modulation of key cellular cascades to promote autophagy initiation, that was conserved between species (Supp Fig. 1B, C), did not align with the abnormal accumulation of autophagic vesicles and markers observed in the presence of miR-7, prompting us to explore subsequent mechanisms that might explain this phenotype.

### MiR-7 blocks autophagy flux

**Fig. 3:**
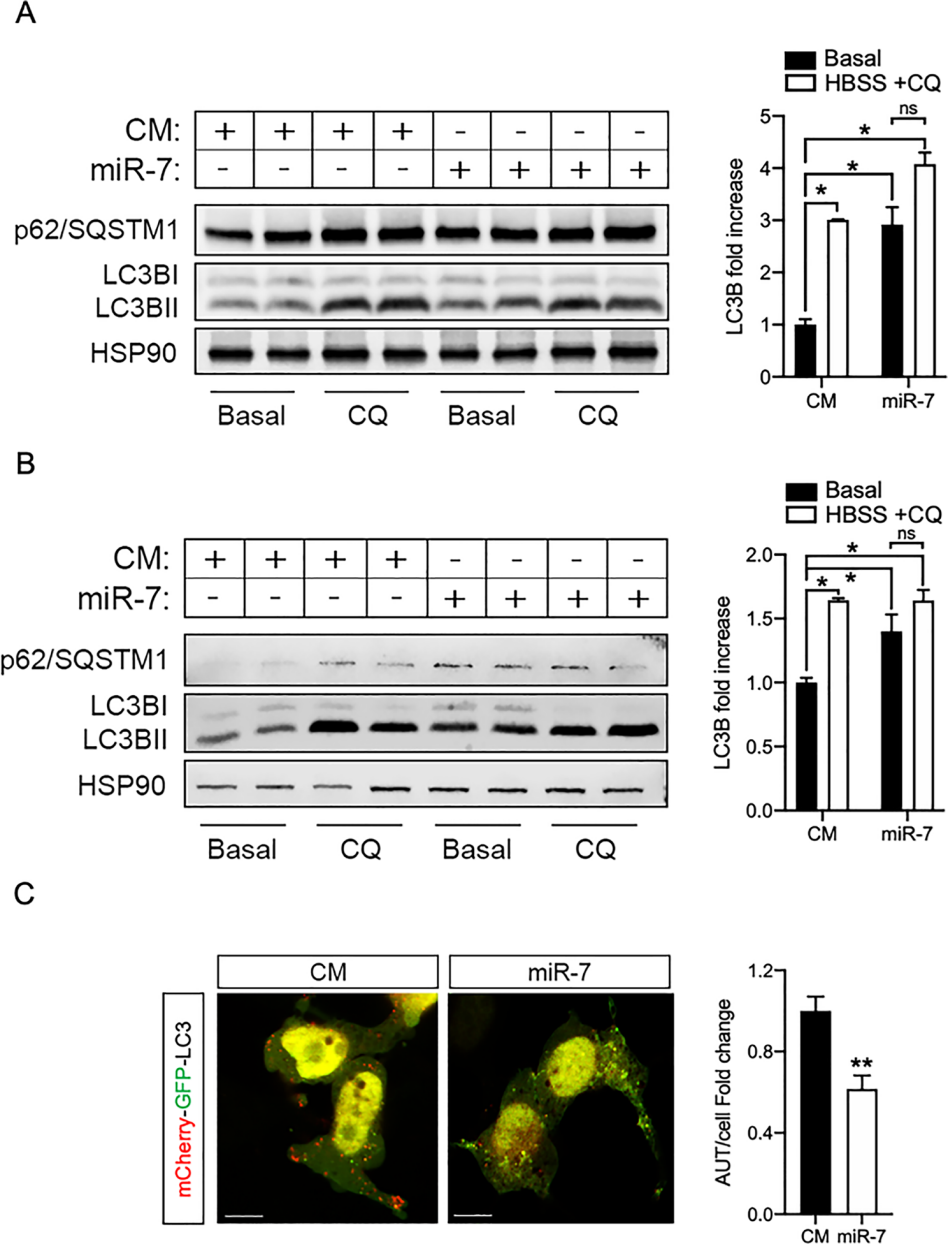
MiR-7 significantly inhibits the autophagic flux. **A-B**. Representative Western Blots of autophagy markers p62 and LC3BI/II and quantification of autophagy flux in SH-SY5Y (**A**) and U87-MG (**B**) cell lines transfected with control mimics (CM) miR-7-5p mimics (miR-7). Cells were treated with 200nM of CQ or vehicle for 6 h. HSP90 was used as loading control. Right panel shows the quantification of LC3BII/I ratio normalized by HSP90 (scaled to 1). Data represents means ± SEM. *P <0.05, significantly different from CM cells. ns, not significantly different. **C**. Measurement of autophagy flux in CM and miR-7 overexpressing U87-MG cells co-transfected with the mCherry-GFP-LC3 tandem reporter. Merged images of two representative cells per condition and quantification of the average number of autolysophagosome (Cherry^+^ and GFP^-^ puncta) per cell, (*n*=3 cells per field, 6 fields per experiment). Data represent the mean of three individual experiments ± SEM. **P <0.01 compared with CM (normalized to 1). Scale bar: 5 μm

Considering these seemingly divergent results, we decided to conduct an autophagic flux assessment in vitro. For these experiments, we starved SH-SY5Y and U87-MG cells with HBSS to activate autophagy and used CQ to purposely block the resolution phase and compared the accumulation rate of LC3B between CM and miR-7 overexpressing cells [29]. As expected, CM transfected cells treated with CQ showed increased levels of p62 and LC3B-II proteins compared to untreated cells (basal conditions), indicating a blockage of autophagic flux. Furthermore, the overexpression of miR-7 in basal conditions was enough to cause significant accumulation of p62 and LC3B, with only a slight additional increase observed upon CQ treatment. Therefore, the analysis of the LC3B/HSP90 ratio in CQ-treated cells vs basal non-treated cells revealed that miR-7 promoted lesser accumulation rate than in CM-cells despite stimulated initiation (Fig. 3A, B), compatible with an inhibition of the autophagic flux. These results were further corroborated using mCherry-GFP-LC3 tandem plasmid where miR-7 overexpressing cells [30] showed a significant decrease in red puncta, indicative of reduced conversion of autophagic vesicles to autolysosomes (Fig. 3C).

### MiR-7 disrupts lysosomal content and function

**Fig. 4:**
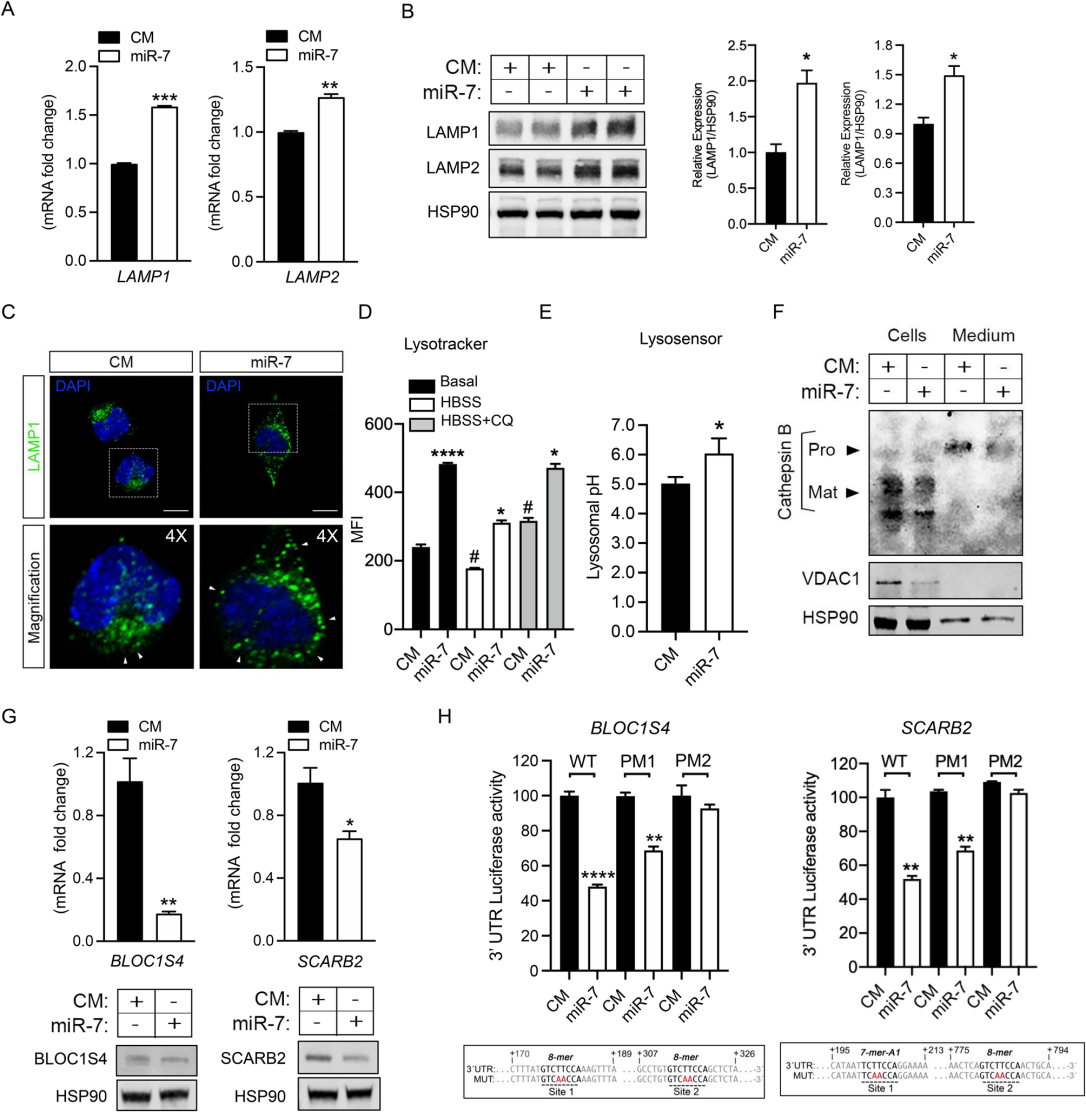
MiR-7 induces the accumulation of defective lysosomes. **A**. qRT-PCR analysis of LAMP1 and LAMP2 mRNA expression levels in U87-MG cells overexpressing control mimic (CM) or miR-7-5p (miR-7). Data are expressed as relative expression levels and correspond to the means ± SEM from three independent experiments performed in triplicate. *P < 0.05, **P < 0.01, ***P < 0.001, significantly different from cells transfected with CM and normalized to 1. **B**. Representative Western blot of LAMP1 and LAMP2 in U87-MG cells transfected with CM or miR-7. HSP90 was used as a loading control. Right panels show relative protein expression normalized to that of HSP90. Data correspond to the means ± SEM of three experiments performed in triplicate. *P < 0.05, significantly different from cells transfected with CM (normalized to 1). **C.** Immunofluorescence confocal imaging of U87-MG cells transfected with CM or miR-7, exposing the punctuated distribution of lysosomes marked with a LAMP1 antibody (green). Nuclei were stained with DAPI (blue). Bottom panels show 4X magnification of the insets. Scale bar: 5 μm. **D.** Determination of lysosomal content by flow cytometry in cells transfected with CM or miR-7 and cultured in basal conditions or nutrient-deprived (HBSS) alone or with CQ at 200 nM for 2 h (HBSS + CQ). Data represent means of three independent experiments ± SEM. *P < 0.05, ****P < 0.0001, significantly different from cells in control conditions. ^#^*P* < 0.05, significantly different from CM in basal conditions. (MFI, Mean Fluorescence Intensity). **E**. Determination of lysosomal pH with LysoSensor Yellow/Blue DND-160 probe in cells transfected with CM or miR-7. Data represent means of three independent experiments ± SEM. *P < 0.05, significantly different from CM cells. **F**. Representative Western blot of cathepsin B, showing its precursor, pro-cathepsin B and the mature form in U87-MG cells or in the culture medium after transfecting CM or miR-7. VDAC1 was used as a positive control of miR-7´s transfection efficacy and HSP90 as a loading control. **G**. qRT-PCR analysis and representative Western blot of BLOC1S4 (left panel) or SCARB2 (right panel) in cells treated as in A. HSP90 was used as a loading control. Data are expressed as relative expression levels and correspond to the means ± SEM from three independent experiments performed in triplicate. *P < 0.05, **P < 0.01, significantly different from cells transfected with CM and normalized to 1. **H**. Luciferase reporter activity in HEK293 cells transfected with CM or miR-7 and the hBLOC1S4 (left panel) or hSCARB2 (right panel) 3′UTR WT or the constructs containing the indicated point mutations (PM) (lower panels, highlighted in red). Data are expressed as relative luciferase activity compared with the activity in control samples cotransfected with an equal concentration of CM and correspond to the means ± SEM of three experiments performed in triplicate. **P < 0.01, ****P < 0.0001, significantly different from cells cotransfected with CM and the WT or PM vectors of the 3′UTRs

Next, we sought to investigate whether miR-7 might impair autophagic flux by targeting the later stages of the autophagy process. One of the potential mechanisms could be lysosomal dysfunction, that would lead to improper degradationof the autophagosome and its content [31]. Indeed, our previous bioinformatic analysis (Fig. 1) revealed targets of miR-7 related to lysosomal biology. To investigate this possibility, we decided to explore lysosomal content and function. First, we assessed the expression of lysosomal markers LAMP1 and LAMP2 by qPCR and Western blot, which showed a significant increase in miR-7 transfected cells compared to control cells (Fig. 4A, B). Also, immunofluorescence staining of cells transfected with miR-7 showed increased LAMP1-marked lysosomes compared to control cells (Fig. 4C), which also correlated with a significantly higher lysosomal mass assessed by lysotracker and flow cytometry (Fig. 4D). In line with these results, mRNA levels of TFEB and TFE3, involved in the transcriptional regulation of lysosome biogenesis [32], were also significantly upregulated in miR-7 overexpressing cells (Suppl Fig. 3A). Despite this, confocal images showed a mislocalization of the lysosomal compartment towards the cell periphery in miR-7 transfected cells, tipically associated with less acidic environment and potential defective functionality. Correlating with this, we found increased lysosomal pH levels in miR-7 overexpressing U87-MG cells compared with control transfected cells (Fig. 4E, Suppl Fig. 3B). Next, we performed Western blot analysis to examine the expression of cathepsin B (CTSB), a cysteine protease essential for lysosomal degradative function and autophagy [33]. As expected, we found that in CM transfected cells, most of the cathepsin B occurred as the mature form, with small amounts of the precursor forms (Fig. 4F). We also observed the precursor forms in the conditional medium isolated from CM transfected cells (Fig. 4F). In contrast, miR-7 overexpression decreased the amount of all forms of intracellular cathepsin B, as well as the extracellular content (Fig. 4F), suggesting that miR-7 compromised the enzymatic properties and the functionality of these organelles. Moreover, consistent with this dysfunctional lysosome phenotype, we found that miR-7 negatively regulates the mRNA and protein expression levels of several key lysosomal targets such as BLOC1S4 and LIMP-2/SCARB2 by directly binding to their 3’UTR as shown by luciferasereporter assay (Fig. 4G, H). In fact, BLOC1S4 is an essential protein for lysosomal biogenesis involved in the trafficking of specific cargo from endosomes to lysosome-related organelles and the proper sorting of membrane proteins or enzymes required for lysosomal function [34]. For its part, SCARB2 is an integral lysosomal membrane protein that regulates the transport of sphingolipids and cholesterol to the lysosomes, which is crucial for maintaining lysosomal membrane stability and ensuring lysosomes functionality [35]. Collectively, the results shown in this section are consistent with a potential role of miR-7 inducing defective lysosomes that could compromise the formation of autolysosomes and therebyinterfering wth the completion of the autophagy process.

### MiR-7 posttranscriptionally regulates the expression of STX17 and SNAP29 and impairs autophagy resolution

**Fig. 5:**
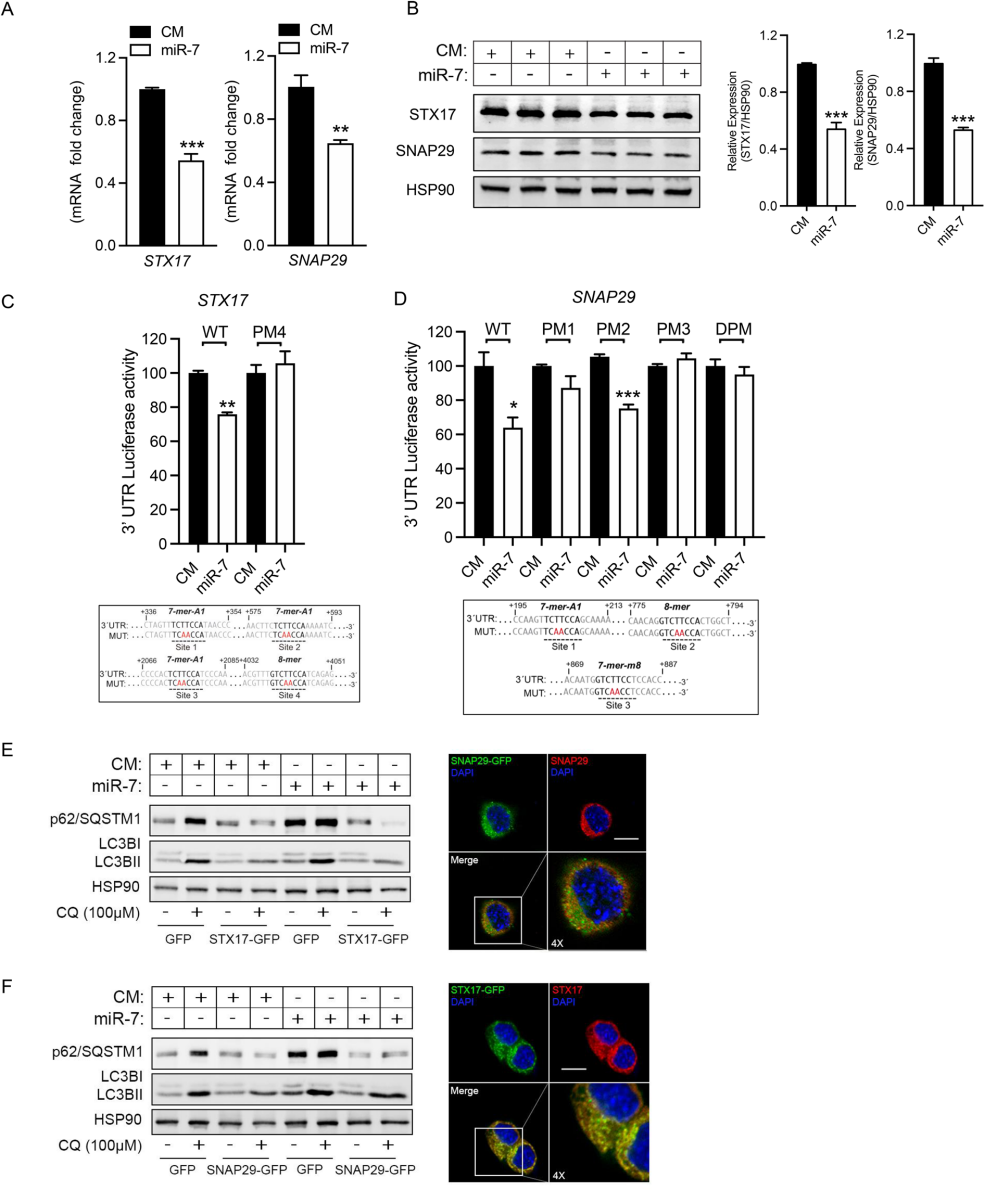
MiR-7 posttranscriptionally regulates the expression of the SNARE complex proteins STX17 and SNAP29. **A**. qRT-PCR analysis of hSTX17 and hSNAP29 mRNA expression levels in U87-MG cells overexpressing control mimic (CM) or miR-7-5p (miR-7). **B**. Representative Western blots of hSTX17 and hSNAP2 in cells treated as in A. HSP90 was used as a loading control. Right graph shows relative protein expression normalized to HSP90. Data correspond to the means ± SEM of three experiments performed in triplicate. ***P < 0.001, significantly different from cells transfected with CM (normalized to 1). **C**. Luciferase reporter activity of STX17 3’UTR in HEK293 cells transfected with CM or miR-7 and with 3’UTRs with wild-type (WT) or the constructs containing the indicated point mutations (PM) or double point mutations (DPM) (upper panels, highlighted in red). Data are expressed as relative luciferase activity compared with the activity in control samples cotransfected with an equal concentration of CM and correspond to the means ± SEM of three experiments performed in triplicate. **P < 0.01, significantly different from cells cotransfected with CM and the WT or PM or DPM vectors of the 3′UTRs. **D**. Luciferase reporter activity of SNAP29 3’UTR as indicated in C. *P < 0.05, ***P < 0.001. **E-F**. Rescue of autophagy flux blockade induced by miR-7. Representative Western blot of autophagy markers p62 and LC3BI/II in cell transfected with CM or miR-7 and a control GFP plasmid or a plasmid expressing STX17 (**E**, STX17-GFP) and SNAP29 (**F**, SNAP29-GFP) ORF regions lacking their 3’UTR. Cells were treated with 200nM of CQ for 6 h. HSP90 was used as loading control. (**E**,** F**, right panels). Immunofluorescence confocal imaging cells transfected the STX17-GFP (**E**) and SNAP29-GFP (**F**) plasmids showing colocalization with endogenous STX17 or SNAP29 (red). Dapi (blue) was used to stain the nucleus. Scale bar: 10 μm

Besides the modulation of the lysosomal compartment, we decided to further explore several other predicted targets of miR-7 involved in the latest steps of autophagy, which could additionally contribute to the impaired phenotype induced by this miRNA. From them, we were particularly interested in STX17 and SNAP29, two proteins that, together with vesicle-associated membrane protein 8 (VAMP8), comprise a SNARE complex that controls the fusion of autophagosomes and lysosomes during the resolution phase of autophagy [36]. Both the autophagosomal member of the complex STX17, and SNAP29, contain a noteworthy number of predicted binding sites for miR-7 in their respective 3’UTR regions (Suppl Fig. 1A), suggestive of regulation by miR-7. Indeed, qPCR and Western blot analyses showed that miR-7 overexpressing cells reduced levels of STX17 and SNAP29 (Fig. 5A, B). Next, the examination of miR-7 effect on their 3′UTRs, performed through a luciferase reporter assay showed that miR-7 significantly repressed hSTX17 and hSNAP29 3′UTR activity (Fig. 5C and D, respectively). Conversely, the mutations of the specific target sites on their 3′UTR blunted the inhibition exerted by miR-7, which further demonstrates the direct interaction of miR-7 with their mRNAs. Next, to define the real contribution of STX17 and SNAP29 in regulating miR-7-induced autophagy blockage, we transfected HEK293 cells with vectors encoding green fluorescent protein (GFP) or STX17-GFP or SNAP29-GFP (Fig. 5E, F, respectively) that lacked the 3′UTRs and therefore, were resistant to the inhibitory action of miR-7. These experiments showed that the blockade of autophagy flux induced by CQ was attenuated by the overexpression of STX17-GFP or SNAP29-GFP, compared to cells transfected with the GFP plasmid under CM conditions. Importantly, the alleviation of the autophagy blockage upon STX17-GFP or SNAP29-GFP overexpression was also observed in the presence of miR-7, effectively rescuing the phenotype. Overall, our data support a dual role for miR-7 in regulating autophagy by one hand promoting the initiation throuth the PI3K/mTOR pathway, while in other hand impairing autophagy resolution by posttranscriptionally targeting STX17 and SNAP29 (Fig. 9A).

### MiR-7 interferes with energetic metabolism by inhibiting Glycolysis and mitochondrial function

**Fig. 6:**
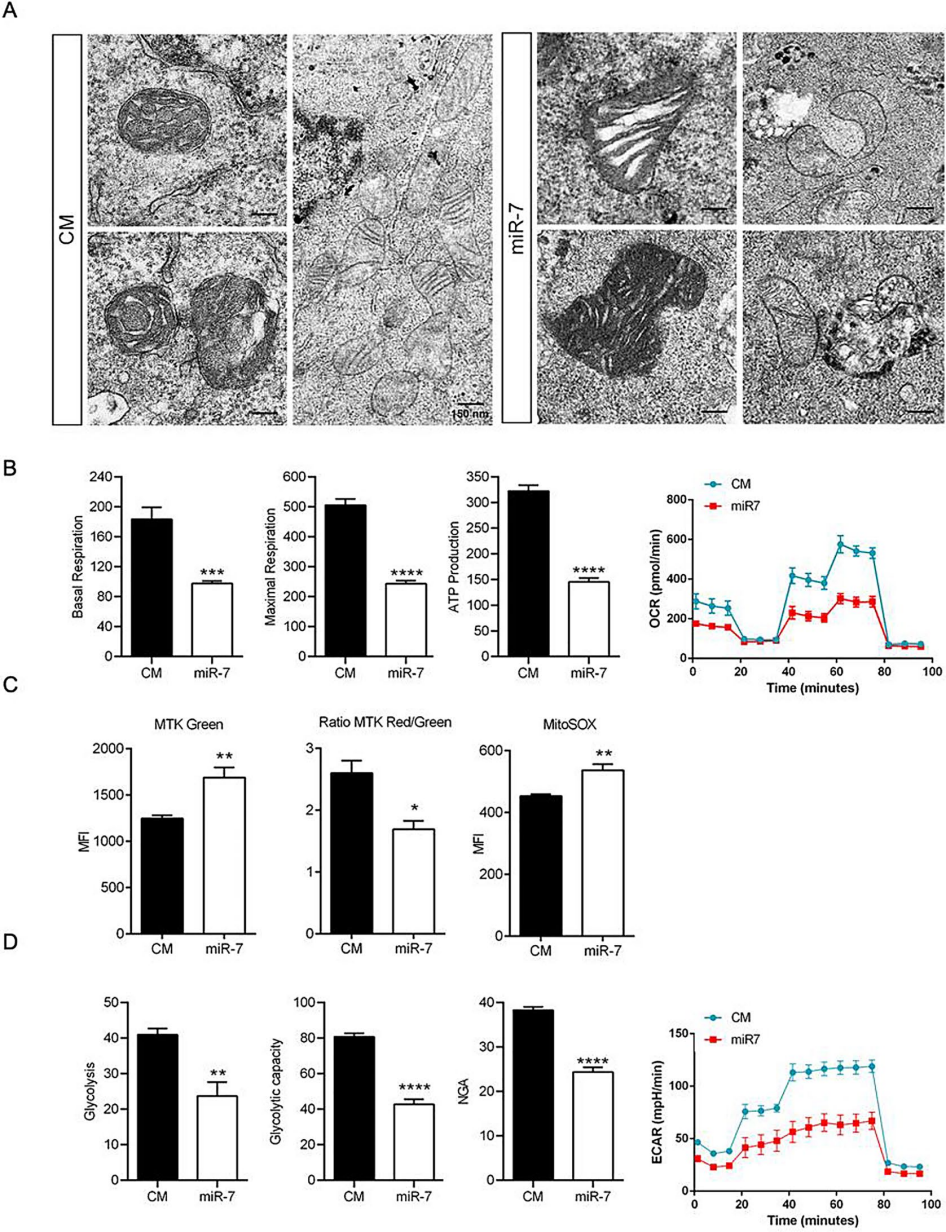
MiR-7 induces mitochondrial dysfunction and energetic stress in vitro. **A**. Representative electron microscopy images detailing the state of mitochondria in control mimic conditions (CM) vs. miR-7-5p mimics (miR-7) overexpression. Scale bar: 150 nm. **B**. Analysis of the mitochondrial respiratory function (basal respiration, maximal respiration and ATP production) under CM vs. miR-7 overexpression via Seahorse through determination of oxygen consumption rate (OCR). Data correspond to the means ± SEM of three experiments performed in quintuplicate. *P < 0.05, significantly different from cells transfected with CM (normalized to 1). **C**. Determination of mitochondrial mass (MTK Green), function (Ratio MTK Red/Green) and superoxide production (MitoSOX) by flow cytometry. Data represent means ± SEM of three experiments performed in quintuplicate. (MFI, Mean Fluorescence Intensity). **D**. Analysis of the glycolytic function (glycolysis, glycolytic capacity and non-glycolytic acidification [NGA]) under CM or miR-7 overexpression via Seahorse through determination of extracellular acidification rate (ECAR). Data correspond to the means ± SEM of three experiments performed in quintuplicate. *P < 0.05, significantly different from cells transfected with CM (normalized to 1). *P < 0.05, ** *P <* 0.01, ***P < 0.001, **** *P* < 0.0001, significantly different from control conditions

It has been already established that GBM cells utilize autophagy as a protumor and survival mechanism against starvation conditions and to eliminate of chemotherapy drugs or accumulation of defective organelle [6]. Mitophagy is a specific type of autophagy fundamental for removing damaged mitochondria via autolysosomes. Given that miR-7 impairs formation of the latest, we hypothesize that it could also interfere with mitochondrial fitness and energetic metabolism. Indeed, the ultrastructural analysis of mitochondria showed that miR-7 promotes significant deformities including swollen cristae, irregular shape, aggregation, and fusion/fission events, in contrast to the rounded and well-formed appearance of mitochondria in CM conditions (Fig. 6A). These results were consistent with an impairment of mitochondrial renewal by miR-7 due to defective mitophagy (Suppl Fig. 4A). In agreement, our experiments showed that mitochondrial respiratory function was also altered by miR-7 (Fig. 6B). Thus, cells that overexpressed miR-7 presented a significant reduction in basal and maximal respiration (≈ 50%) ATP production, when compared with control conditions, which corroborated the impact of this miRNA on the cell energetic state. Additionally, we assessed mitotracker green staining (MTK Green) (Fig. 6C) and mtDNA quantification (Suppl Fig. 4B), which showed a significant increase of mitochondrial mass (mt mass) in the presence of miR-7. In line with this, qPCR analysis revealed a significant upregulation of genes involved in mitochondrial biogenesis and remodeling, such as PGC1α, NRF1, Sirt1 and MFN1 and MFN2. while genes related to mitochondrial function, including SDHC and VDAC1, were significantly lower upon miR-7 overexpression (Suppl Fig. 4C). Despite that, decreased ratio of MTK Red, an indirect marker of negative mitochondrial potential (Δψm), versus MTK Green, suggested that the augmented mitochondrial population in miR-7 overexpressing cells, had less Δψm, reflecting the incapacity to maintain mitochondrial potential and correlating with the reduced oxidative capacity observed in the presence of this miRNA. Our analysis also showed that miR-7 increased the production of superoxide reactive species (MitoSOX staining), which represents another sign of poor mitochondrial performance (Fig. 6C). Finally, although not carried out by the mitochondria, our experiments also demonstrated that miR-7 significantly hinders glycolysis and the glycolytic capacity (Fig. 6D), which indirectly affects the supply of pyruvate—the initial metabolite of the tricarboxylic acid cycle (TCA cycle) necessary for producing NADH required for oxidative phosphorylation, that ultimately can contribute to a decrease in the metabolic capacity of the cell. We further characterized this glycolytic impairment by identifying several key enzymes as putative miR-7 targets, including ALDO1, PGK1, ENO2, and PDK1 (Suppl Fig. 5A, B). Subsequent qPCR analyses showed a significant reduction in mRNA levels of ALDO1, ENO2, and PDK1 (Suppl Fig. 5C). Among these genes, ENO2 represented a particularly relevant target due to its established prognostic value in GBM (not shown). Importantly, our analysis confirmed a marked reduction of ENO2 protein levels in U87-MG cells overexpressing miR-7 (Suppl Fig. 5D). Notably, both mRNA and protein expression of ENO2 were strongly suppressed in miR-7 overexpressing GBM tumors (Suppl Fig. 5E, F). Finally, luciferase reporter assays confirmed that miR-7 significantly inhibits the ENO2 3′UTR (Suppl Fig. 5G). In summary, these findings underscore a critical role for miR-7 in repressing glycolysis, which, together with its effects on mitochondrial function, leads to a profound disruption of cellular bioenergetic capacity.

### MiR-7 inhibits tumor growth in a GBM xenograft model

**Fig. 7:**
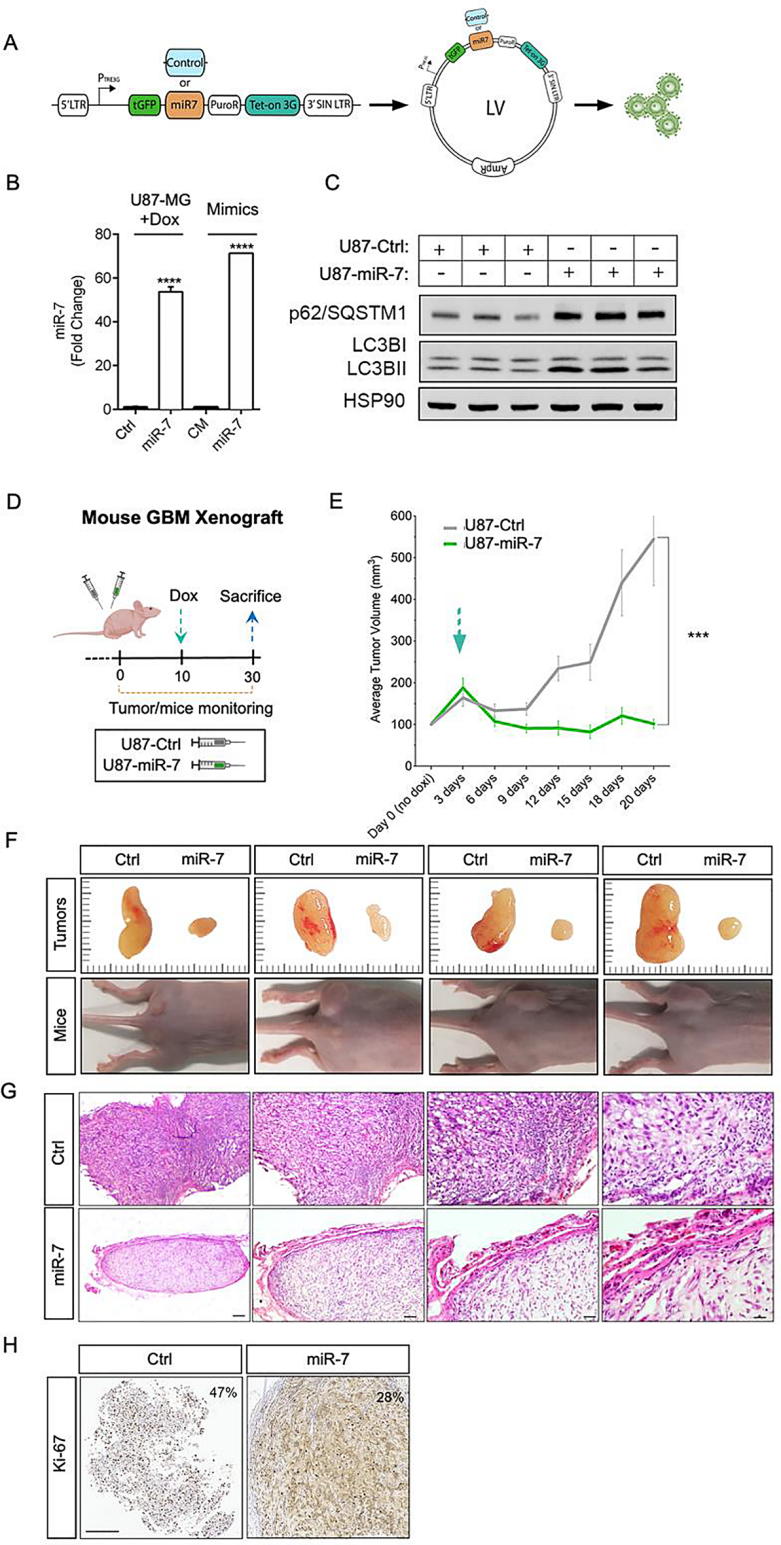
MiR-7 blocks tumor growth in a GBM xenograft model. **A**. Schematic representation of the procedure used to generate stable U87-MG Ctrl (U87-Ctrl) and miR-7 (U87-miR-7) cell lines, through lentiviral infection and selection, capable to stably express GFP and a control scramble miR (Ctrl) or miR-7 after induction with doxycycline (Dox). **B**. RT-qPCR of mature miR-7 in U87-Ctrl and U87-miR-7 cells after Dox induction and in U87-MG cells transfected with control (CM) and miR-7-5p mimics (miR-7). Data represent means ± SEM of three experiments performed in triplicate. ****P < 0.0001, significantly different from control conditions. **C**. Representative Western Blot of autophagy markers p62 and LC3BI/II in U87-Ctrl and U87-miR-7 cell lines after 48 h of Dox treatment. HSP90 was used as loading control. **D**. Timeline of the GBM xenograft experiment performed. **E**. Average volume of tumors over the time of the experiment. Blue arrow represents the time of induction of expression with Dox treatment. **F**. Tumor size comparison of Ctrl and miR-7 tumors beside their corresponding mouse. Lines at the sides represent a ruler (1 mm between short lines, 5 mm between long lines). **G**. Representative images of the H&E staining of Ctrl and miR-7 tumors at different amplifications (5-40X). Scale bars: 800/400/200/100 µm. **H**. Representative images of Ki-67 immunostaining in Ctrl and miR-7 tumors. Percentage of Ki-67-stained nuclei is noted in the right upper corner (H-Score). Scale bar: 200 μm

Once we have demonstrated the detrimental effect promoted by miR-7 on autophagy and energetic metabolism in GBM, we proceeded to explore whether these alterations could contribute to its antitumor actions in a mouse GBM xenograft model. While a previous work reported a role of miR-7 in the initiation and progression of the tumor by inhibiting the proliferative pathways PI3K or Ras [37], we took a step further interrogating miR-7 effects on GBM in already formed tumors, which resembles a more realistic setting of the disease in these patients. To do so, we used a lentiviral-mediated doxycycline (Dox)-inducible system to generate a U87-miR-7 stable cell line that overexpressed miR-7 on demand under timely-control (Fig. 7A). Our analysis showed that after 48 h of induction with Dox, miR-7 expression was significantly increased compared to the U87-Ctrl cells, to the same extent of cells transfected with miR-7 mimics (Fig. 7B). Moreover, U87-miR-7 cells showed accumulation of autophagy markers LC3B and p62 compared to U87-Ctrl cells (Fig. 7C), recapitulating autophagy impairment observed before. Similar results were found in tumorsphere assays, where miR-7 strikingly reduced the cellularity of these 3D structures by a slight cell cycle arrest (Supp Fig. 6). Next, to perform with the in vivo xenograft model, ten immunocompromised nude mice (ATHYM-Foxn1^nu/nu^) were subcutaneously injected with U87-Ctrl cells or U87-miR-7 cells in alternating flanks between animals to reduce mice and injection-side bias. Nine of ten mice survived until the end of the experiment and no significant changes were observed on mice weight (data not shown). The experimental timeline is detailed in Fig. 7D, where tumor size was monitored along the experiment to track tumor progression. When established tumors were observed after ten days of cell inoculation, miR-7 expression was induced in vivo with Dox. As expected, control tumors kept growing at a high rate, reaching an average size superior to 500 mm^3^. Strikingly, miR-7 tumors’ growth not only stalled but their size was even reduced, staying at approximately 100 mm^3^ until sacrifice (Fig. 7E). The significant reduction in tumor volume and size upon miR-7 overexpression is illustrated in the images shown in Fig. 7F. Additionally, Ctrl tumors seemed to have higher vascularization than miR-7 ones, where capillaries were undetectable (Fig. 7F). Further histological characterization of the specimens revealed lesser cellularity and the presence of an encapsulation layer in miR-7 tumors that was absent in Ctrl tumors, which showed irregular borders instead (Fig. 7G). In addition, small nuclei were spotted in Ctrl tumors suggestive of infiltrating immune cells, along with red blood cells, while in miR-7 overexpressing tumors, red blood cells were only found in a limited area close to the capsule (Fig. 7G). Next, Ki-67 immunostaining revealed a decreased proliferation rate in miR-7 tumors, which correlates to the cycle cell arrest observed in vitro (Supp Fig. 6) and was also accompanied by a significant reduction in the H-Score (Fig. 7H). Additionally, miR-7 overexpressing tumors resulted in mitochondrial deformities (Suppl Fig. 7A) and the accumulation of intracellular vesicles, as observed through EM analysis and elevated LC3B- and LAMP1-positive immunostaining compared to Ctrl tumors, thereby corroborating previous findings (Suppl Fig. 7B, C, respectivelly).

### MiR-7 promotes changes in ECM remodeling in GBM

**Fig. 8:**
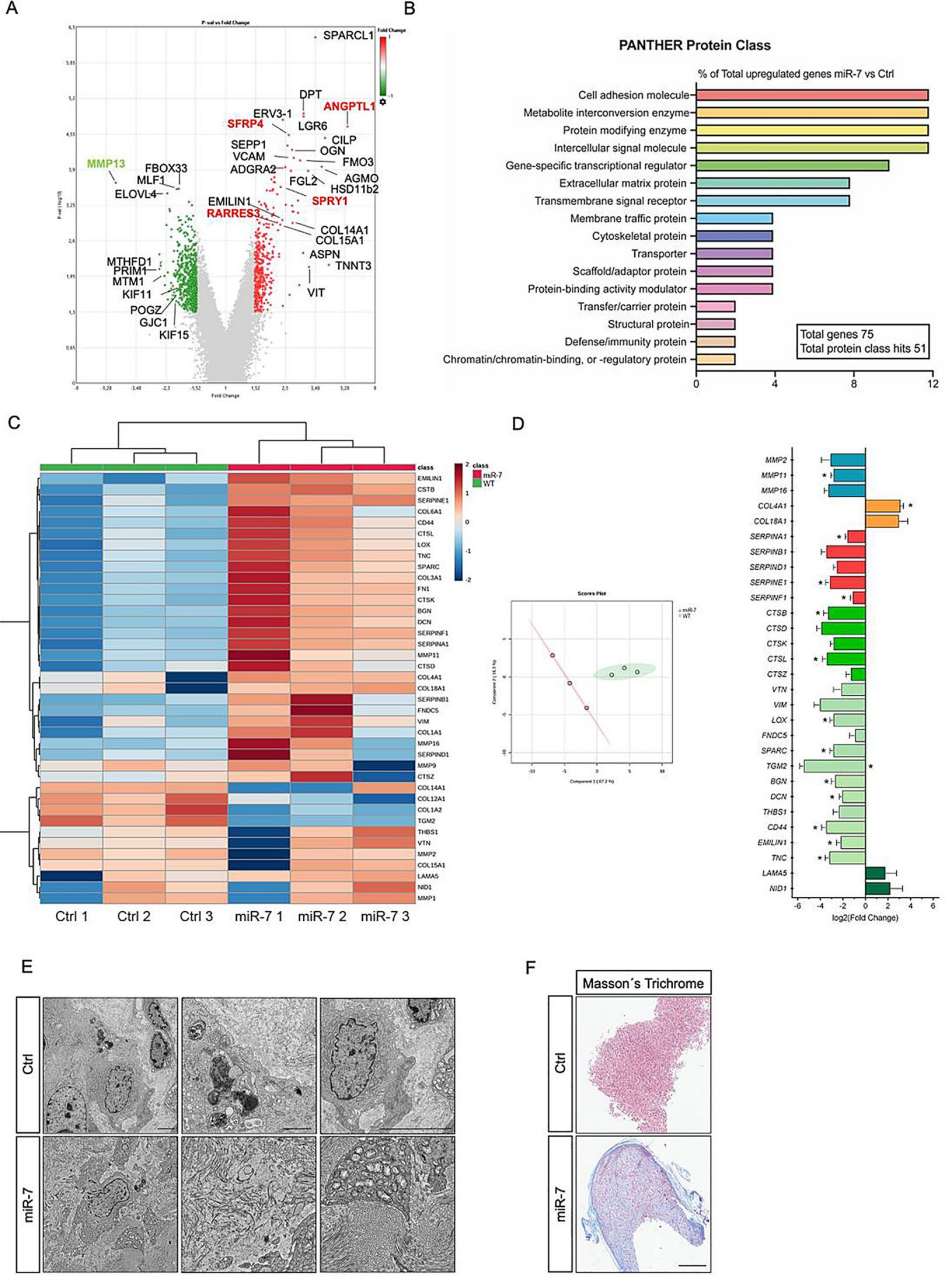
MiR-7 alters the expression pattern of GBM tumors and promotes ECM remodeling. **A**. Volcano plot representation of upregulated (red) and downregulated (green) genes in the genome-wide transcriptomic analysis from Ctrl and miR-7 GBM tumors. **B**. GO analysis of the upregulated genes in PANTHER software. **C**. Matrisome analysis. Heat map representation of the transcriptomic analysis of the upregulated (red) and downregulated (blue) ECM genes in Ctrl (green) and miR-7 (magenta) tumors, with a hierarchical diagram of the samples on the top. **D**. Principal component analysis generated using 48 genes of matrisome panel in three Ctrl tumors and three miR-7 samples (left panel). Expression analysis of selected ECM genes (right panel). *P < 0.05, significantly different from Ctrl tumor samples. **E**. Electron microscopy images of Ctrl and miR-7 tumors to visualize the extracellular space. Scale bar: 5 μm. **F**. Images of Masson’s Trichrome staining of Ctrl and miR-7 tumors. Collagen appears in blue. Scale bar: 800 μm

The remarkable effect of the miR-7 restoration on reducing GBM oncogenic features, combined with the histological features observed in these tumors and the multifaceted roles of miRNAs, able to simultaneously influence various cellular pathways, led us to investigate additional mechanisms underlying the anti-oncogenic action of miR-7 in GBM. To this end, we conducted a comprehensive transcriptomic analysis in GBM tumors. This approach showed a differential expression pattern between control and miR-7 tumors (Fig. 8A), that were subsequently analyzed for functional classification (Fig. 8B). Results obtained from downregulated genes in miR-7 tumors subjected to GO analysis showed a significant enrichment of protein-coding genes involved in functions such as cell adhesion, cytoskeletal remodeling and ECM (Fig. 8B). According with these results, one of the most downregulated genes corresponded to matrix metalloproteinase (MMP)-13 (MMP13), highly expressed in invasive stem-like GBM cells [38] and plays an important role in tumor aggressive process by degrading ECM. Several other interesting genes were downregulated in miR-7 tumors such kinesin-12, also known as KIF15, a microtubule-dependent motor protein that also participates in mitosis [39], another mitotic kinesins such as KIF11, a current therapeutic target against GBM to block invasion, proliferation, and self-renewal [40]. This correlates with in vitro results showing that miR-7 impairs migration and invasion capacities (Suppl Fig. 8). However, none of these genes contain predicted binding sites for miR-7, ruling out a direct regulation. By contrast, among the genes that showed the highest upregulation in our array we found angiopoietin-like 1 (ANGPTL1), a well-known anti-angiogenic gene normally reduced in GBM [41], the secreted frizzled-related protein 4 (SFRP4), a Wnt antagonist also reduced in high grade GBM that helps eliminate glioma cells by making them more vulnerable to chemotherapy [42], RARRES3 which is a tumor suppressor gene also inhibited in advance cancers [43], or Asporin (ASPN), a small leucine-rich repeat proteoglycan, identified as a growth suppressor of GBM cells in vitro and in vivo [44]. These findings, together with the differential morpho-histological features found before suggested that remodeling of the ECM in GBM tumors could represent a new potential anti-oncogenic mechanism of miR-7. Considering the growing interest in ECM remodeling within the cancer field [45], the limited understanding of its role in GBM and potential regulation by miRNAs, we further investigated these observations in vivo. In order to characterize and validate miR-7 targets involved in ECM, we performed a secondary analysis applying a sequential selection strategy among significantly downregulated transcripts in miR-7 tumors, based on the following criteria: (i) confirmed ECM-related function; (ii) prognostic relevance and correlation with tumor size reduction; and (iii) predicted miR-7 sites within the 3′UTR (Suppl Fig. 9A). This approach identified LGALS8 (Galectin-8), a glycan-binding protein involved in ECM–integrin interactions, which is frequently upregulated in GBM and associated with poor prognosis by promoting adhesion, migration, proliferation, and apoptosis resistance (Suppl Fig. 9B). Consistent with this, qPCR and Western blot confirmed that LGALS8 expression was significantly reduced in miR-7 tumors (Suppl Fig. 9C). Finally, luciferase assays demonstrated that miR-7 significantly inhibits the activity of the 3′UTR of LGALS8, supporting a novel mechanistic link between miR-7 activity and impaired ECM-mediated tumor progression (Suppl Fig. 9D). Additionally, to provide further robustness to our observations, we performed a targeted-transcriptomic matrisome assay by assessing a set of well-known genes to intervene with ECM remodeling (Fig. 8C). Principal components analysis (Fig. 8D, left panel) reflected clear differences between GBM tumors and provided strong evidence supporting that miR-7 overexpressing tumors exhibit a remarkably altered matrisome. Indeed, the comparative analysis in the matrisome assay recapitulated some of the regulated targets previously observed, including EMILIN1 (elastin microfibril interfacer 1) and CTSB or SPARC (secreted protein acidic and cysteine rich) (Fig. 8D, right panel). Differential changes in the expression of these genes were then represented based on their function to better visualize potential effect of miR-7 on ECM remodeling. The results shown in Fig. 8D (right graph) indicated an overall downregulation of metalloproteinases (MMPs) and members of two distinct families of proteases—serpins and cathepsins—along with several other genes, including TGM2, a transglutaminase that catalyzes the crosslinking of extracellular proteins and CD44, a receptor for hyaluronic acid (HA) that also interacts with collagens and MMPs. Additionally, Decorin (DCN), which plays a role in collagen fibril assembly, and Tenascin-C (TNC), commonly expressed in gliomas, were also downregulated. By contrast, two families of genes include Laminin alpha-5 (LAMA5) and Nidogen 1 (NID), that mediate cell attachment and migration, were upregulated but did not reach statistical significance.

Next, we performed a detailed examination of tumor ultrastructure by EM (Fig. 8E), which revealed the presence of long electron-dense fibers of collagen that occupy a great portion of the extracellular space of the miR-7 tumors compared to Ctrl tumor sections. Next, Masson’s Trichrome staining confirmed that these fibers in miR-7 tumors were, at least partially, made of collagen (Fig. 8F). Overall, these findings reveal previously unknown mechanisms by which miR-7 alters ECM composition, potentially promoting tumor encapsulation, restricting cell movement, and thereby inhibiting GBM growth.

## Discussion

GBM is one of the most challenging neuro-oncological conditions to treat due to the current lack of effective curative therapies. GBM is characterized by multiple cell strategies that allow its rapid adaptation to challenging environments that favor its progression. These include genetic mutations in major metabolic drivers, such as EGFR and other proliferative pathways [11] or others that contribute to the establishment of the characteristic switch to aerobic glycolysis, also known as the Warburg effect [46]. Besides, epigenetic alterations mediated by miRNAs have been shown to contribute to the physiology of GBM [47], by acting on the already described genes and pathways (EGFR, PI3K, etc.) and other key cellular processes essential for tumor survival. Although these reports include miR-106, miR-143, miR-326 and let-7a, the interference on GBM seems to be at a single level by affecting glycolysis or energy metabolism [11]. Of particular interest are those miRNAs that are highly represented in the central nervous system or oscillating in the brain under normal versus pathological conditions, such as miR-7, having a higher chance to play a pathophysiological role or to intervene in specific cellular processes [48, 49]. Indeed, miR-7 has been found to be differentially expressed in multiple cancers. In the brain, this molecule is abundant under normal conditions where it plays a critical role during brain development and neuronal differentiation [50]. However, miR-7 is abnormally elevated in neurological disorders such as Alzheimer’s disease [13], whereas, by contrast, its levels are notably suppressed in GBM, supporting its tumor-suppressive role in this pathological context [12]. These fluctuations across developmental and disease states reveals the context-dependent functionality of this miRNA and underscore the importance of this miRNA in the central nervous system and during different brain pathologies. Certainly, previous studies from our group have shown that miR-7 regulates important homeostatic functions in the brain through its targeting of key elements of insulin signaling pathway and cholesterol metabolism, adding even more evidence to its metabolic relevance in the brain [13, 14]. In addition, it is plausible that differential expression in response to specific stimuli, together with the relative abundance of miR-7 and its mRNA targets under distinct metabolic conditions, may influence its final biological outcomes. This complexity is further increased by the fact that miR-7 levels and activity can also be modulated by RNA binding proteins, competing endogenous RNAs, or alterations in miRNA processing pathways, adding additional layers of regulation beyond transcriptional control [51]. In conclusion, distinguishing the physiological roles of miR-7 in the healthy brain from its tumor-suppressive functions remains a complex question that many researchers are actively investigating, and it will require additional experimental work to be fully elucidated.

In this study, we explored novel metabolic functions of miR-7 that may contribute to the physiopathology of GBM, while also identifying these mechanisms as potential therapeutic targets for innovative treatment strategies against this aggressive tumor. Notably, our data show that miR-7 plays a dual role in the autophagy pathway by promoting the initiation of the process while simultaneously causing a blockade at the late resolution stage. These effects result in the accumulation of autophagic vesicles, confirmed by EM analysis in miR-7 overexpressing cells and the significant increase of MDC-positive vesicles, together with an impairment in the autophagy flux. Gene and protein expression analysis further supported these findings by showing significant modulation of regulatory genes associated to lysosomal biology and autophagy function. Mechanistically, we found that the blockade of autophagy by miR-7 lies in lysosomal dysfunction rather than a reduction in lysosomal content. Indeed, we observed an increase in lysosomal mass, detected via LAMP1/2 expression and FACS analysis, even under autophagy induction, along with impairment of lysosomal pH and diminished lysosomal catalytic activity based on CTSB maturation. A deeper analysis of miR-7 targets involved in autophagy and lysosomal functions identified key players such as BLOC1S4 and SCARB2 as direct targets of miR-7. Indeed, human mutations in BLOC1S4 are associated with Hermansky-Pudlak Syndrome, a genetic disorder characterized by defects in lysosome-related organelles, while SCARB2 is necessary for lysosomal membrane integrity [34, 52, 53]. In line with this, it is generally accepted that functional miRNAs that play a significant role in a biological pathway typically target multiple genes within that same pathway [54, 55]. Importantly, further exploration led us to establish STX17 and SNAP29 as direct targets of miR-7. These genes represent critical components of the SNARE complex required for the fusion of lysosomes with autophagosomes, a key step for autophagy resolution. Rescue experiments with miR-7 resistant forms of STX17 and SNAP29 were able to alleviate the autophagy blockade, confirming that these two targets are crucial to the phenotype induced by this miRNA. Concurrent with autophagy disruption, miR-7 overexpression was accompanied by significant defects in mitochondrial morphology and function, materialized by a notable decrease in energy production and increased oxidative stress. All these phenomena, together with impaired glycolytic capacity, supported by the inhibition of several glycolitic enzymes, particularly ENO2 through a direct posttranscriptional regulation, can lead the cells to undergo a starvation-like state, forcing them to remodel mitochondria in a desperate attempt to correct the energetic deficient state, in agreement with a previous report [56]. However, miR-7’s interference with autophagy and mitophagy, which includes reduced recycling of defective mitochondria, may further contribute to mitochondrial and bioenergetic inefficiency in the cell. Previous studies in other biological settings like the endothelium or in lung cancer have corroborated the role of miR-7 blocking autophagy and mitophagy [8, 57]. Putting these results in the context of GBM, we may assume that miR-7 overexpression could be effectively strangling energetically the cell, which may explain why the signaling pathways to initiate autophagy, herein, decreased mTOR signaling and increased AMPK phosphorylation, were activated in our experiments. In recent years, targeting the autophagy-dependent vulnerabilities of cancer cells has gained significant attention, leading to strong support for the use of autophagy inhibitors [58, 59] in conjunction with traditional therapies [60]. The conditional deletion of genes essential for autophagy in the host reduces the availability of metabolic substrates for rapidly proliferating tumor cells, which hinders tumor growth [61]. Additionally, in experiments where autophagy is blocked, cells usually experience an increased sensitization to TMZ, so many groups are examining the effects of autophagy inhibitors in combination with TMZ. However, depending on the step of the process wherein the inhibition is done, effects on TMZ sensitivity could vary. For instance, 3-methyladenine, a blocker of the incorporation of LC3 into the forming autophagosome membrane, does not affect sensitivity to TMZ, while late-stage blockers, such as bafilomycin A1 [62] or CQ [63] have shown benefits. This could be caused by the cytotoxicity that the accumulation of autophagic vesicles induces when treated with bafilomycin A1 and CQ [64], absent in 3-methyladenine treatment since autophagosomes cannot even be formed in the first place. Highlighting the relevance of these findings, our work not only expands on miR-7’s known involvement in autophagy but also underscores its dual effects on both initiation and resolution, which could additionally enhance the efficacy of TMZ treatment or increase the vulnerability of cancer cells. Therefore, these miR-7 novel actions can provide new therapeutic opportunities against GBM, where selective mTORC1/PI3K inhibition has shown promise when combined with the later-stage autophagy inhibitor CQ [65].

Although miR-7 has been shown to reduce tumor growth in GBM through the regulation of EGFR [66], our experimental xenograft approach provides advantages from the translational and scientific point of view: first, its emulates a clinical setting wherein miR-7 induction can be analyzed in already grown tumors, given that in patients they often reach about two centimeters in diameter upon diagnosis [67]; and two: it allowed us to further investigate the biological functions and effects of miR-7´s restoration in vivo, extending beyond proliferation, as it was the case of our previous autophagy and mitochondrial impairment shown in vitro. One of the most striking results found was the significant reduction in tumor size and volume under miR-7 re-expression in mice, ranging from 6 to 10-fold compared to controls, that were more pronounced than previously observed in liver or GBM xenograft models [68, 69]. Along with the expected reduction in proliferation observed through Ki-67 staining, histological differences featured between tumor specimens suggested additional mechanisms by which miR-7 may contribute against GBM. In agreement, transcriptome analysis revealed extensive changes in gene expression related to cell adhesion, cytoskeletal remodeling, and ECM dynamics, highlighting one of the key differences between GBM and normal brain tissue: the ECM composition, which is a major factor influencing tumor aggressiveness and migration [70]. In invasive cancer cell lines, an impairment of migration has been observed when miR-7 is expressed [71, 72]. In agreement with this, our data on defective migration and invasion capacities in glioblastoma cells expressing miR-7 could possibly correlate with our findings showing a reduction in MMPs, CTSs and SERPINs, all linked to ECM degradation [73]. Indeed, simultaneous inhibition of MMP9 and CTSB, both downregulated in miR-7 overexpressed GBM tumors, has already been explored as a potential glioma treatment [74, 75]. The attenuated capacity of degradation of the ECM that could reflect the downregulation of the aforementioned enzymes could significantly increase the stiffness, another characteristic of GBM [5]. However, despite that increased ECM stiffness is commonly associated with a pro-oncogenic profile and vascularization in GBM, this effect was not observed in miR-7 tumors, probably due to the already described inhibitory actions of miR-7 on VEGF expression [76]. An additional result that further supports our in vivo observations in miR-7 tumors is the significant downregulation of LGALS8 expression, which is generally upregulated in GBM and it associates with poor prognosis. This galectin family member plays a pivotal role in modulating ECM–integrin interactions, key elements of the tumor microenvironment, and contributes to tumor aggressiveness by enhancing cell adhesion, migration, proliferation, and resistance to apoptosis [77]. Importantly, inhibition of LGALS8 expression may occur throught a direct posttranscriptional targeting by miR-7, establishing a mechanistic link between miR-7 activity and the suppression of ECM-driven tumor progression.

Another notable difference from previous reports on GBM, which typically show minimal intra-tumoral fibrillar collagen [78], is the specific observation of long bundles of collagen in miR-7 tumors, as demonstrated by ultrastructural analysis and Masson’s trichrome staining. In that line, downregulation of MMPs in the miR-7 tumors could be hindering motility and confining tumor cells to better-defined tumor borders [79].

This reinforced ECM phenotype, together with our evidence on the impact on the autophagic activity by miR-7, points to the potential value of therapeutic strategies that simultaneously target both the ECM and the autophagy–lysosome system. Accumulating evidence indicates the existence of such mechanistical crosstalk, where autophagy regulates ECM turnover and stiffness, while ECM-derived mechanical and biochemical cues modulate autophagic flux [80]. Indeed, a recent study has highlighted that inhibitors of heparanase—an enzyme that modulates the ECM and promotes both a tumor-permissive microenvironment and autophagy activation—show early signs of efficacy in lymphoma [81], althought its role in GBM remains unknown. Other potential ECM components with autophagy-inhibitory functions are the proteoglycans perlecan and lumican, which stood out as slightly but significantly upregulated in miR-7 tumors (not shown), and notably, elevated lumican levels have been linked to improved prognosis and reduced aggressiveness in GBM patients [82]. Nevertheless, despite growing interest in targeting ECM–autophagy crosstalk in cancer, our understanding of these mechanisms in GBM remains incipient. In this emerging field, our data indicate that miR-7 overexpression may recapitulate the effects of such multifaceted intervention, as it simultaneously modulates autophagy-related pathways and ECM remodeling—representing a promising alternative to synergistically impair tumor survival, invasion, and therapy resistance.

Finally, given the therapeutic potential of miR-7 revealed by our study, it is important to acknowledge the translational challenges associated with its clinical application, including potential off-target effects, limited stability, and inefficient delivery across the blood-brain barrier. To address these limitations, several advanced delivery strategies are under development. Among them, nanoparticle-based systems and biocompatible hydrogels have shown promise for achieving localized and sustained release of miRNA mimics directly into the tumor cavity, particularly after surgical resection, thus minimizing systemic exposure and improving tumor specificity [83, 84]. In particular, recent studies have highlighted graphene oxide nanosheets as efficient delivery vehicles for miR-7, demonstrating successful intracellular transfer in GBM cell lines and significant antitumor effects in xenograft models through inhibition of the PI3K/AKT pathway [85]. These emerging technologies may enhance the safety and efficacy of miR-7-based therapies, supporting their future development as targeted interventions in GBM.

Overall, the results presented here provide novel scientific evidence supporting the role of miR-7 in preventing GBM progression, likely by maintaining the tumor in its early stages. This effect may compel the tumor to remain compact and non-vascularized by reducing ECM degradation, while simultaneously promoting ECM remodeling towards a less malignancy-prone profile. Additional research is needed to investigate the role of other key pro-oncogenic genes identified in our study as inhibited targets by miR-7 restoration in GBM tumors, thereby enhancing the multifactorial therapeutic potential of miR-7 against this disease.

**Fig. 9:**
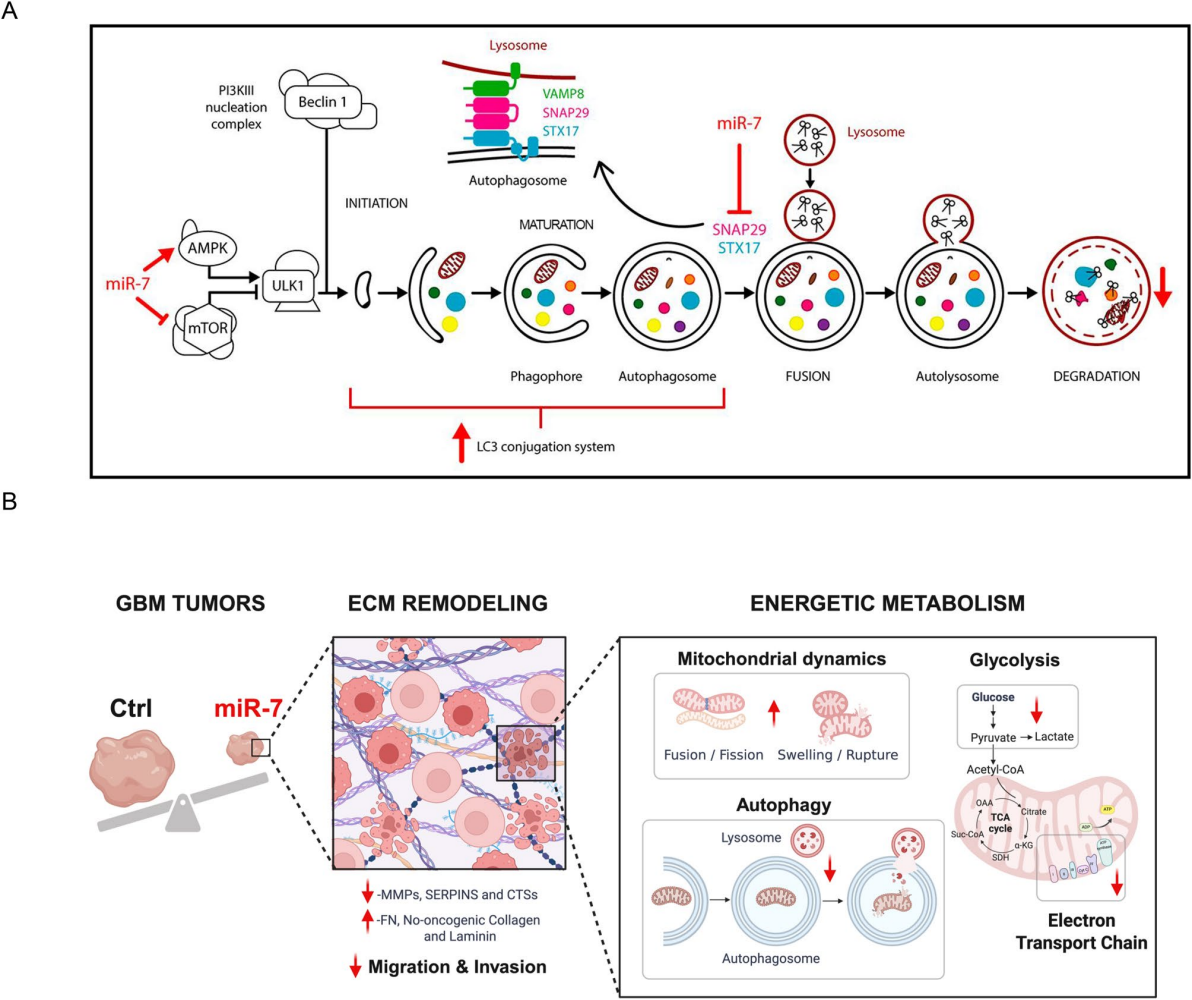
Squematic summary of miR-7 effects on GBM. **A**. Schematic representation of the proposed mechanism of action of miR-7 on autophagy. Red arrows show the steps where miR-7 has its influence or main effect. A detail of the STX17–SNAP29–VAMP8 SNARE complex is shown as well to further illustrate the role of SNAP29 and STX17 in the autophagosome and lysosome fusion. **B.** Schematic representation summarizing the effects miR-7 on GBM tumors, ECM remodeling and energetic metabolism. Red arrows indicate up- or down-regulation of specific functions exerted by miR-7. llustration were created with BioRender (https://biorender.com)

## Conclusion

In summary, the data herein presented provide solid and convincing evidence demonstrating that miR-7 inhibits GBM growth and malignancy progression by targeting key pathways involved in autophagy, energy metabolism, and ECM remodeling (Fig. 9). These findings depict miR-7 as a promising candidate for future therapeutic interventions of GBM, a brain tumor with dreadful survival chances for which there is not yet a definitive cure, therefore offering a clinically relevant opportunity that should be tested for use in humans.

## Supplementary Information

Below is the link to the electronic supplementary material.

Supplementary Material 1

## Data Availability

The datasets generated and analyzed during the current study are not publicly available due to their ongoing use in subsequent publications but are available from the corresponding author on reasonable request.

## Abbreviations

GBM: Glioblastoma multiforme
miR-7: microRNA 7
ECM: Extracellular matrix
EGFR: Epidermal growth factor receptor
PI3K: Phosphoinositide 3-kinase
mTOR: Mechanistic target of rapamycin
AKT: Protein kinase B
AMPK: AMP-activated protein kinase
LC3B: Microtubule-associated protein 1 A/1B-light chain 3
SQSMT1/p62: sequestosome 1
CQ: Chloroquine
SNAP29: Synaptosomal-associated protein 29
LAMP1/2: Lysosome-associated membrane protein 1 /2
ATG: Autophagy-related gene
MMPs: Matrix metalloproteinases
BLOC1S4: Biogenesis of lysosomal organelles complex-1 subunit 4
SCARB2: Scavenger receptor class B member 2
STX17: Syntaxin 17
SNARE: Soluble N-ethylmaleimide-sensitive factor attachment protein receptor
MDC: Monodansylcadaverine
CTS: Cathepsins
TMZ: Temozolomide
Ki-67: Antigen identified by monoclonal antibody Ki-67
MMP9: Matrix metalloproteinase 9
SERPINs: Serine protease inhibitors
