# Supplementary material for: MiR-7 inhibits progression of glioblastoma by impairing autophagy resolution, energy metabolism and ECM remodeling
Source: J Exp Clin Cancer Res. 2025 Aug 14;44:237. doi: 10.1186/s13046-025-03504-6 (PMC12351809; doi:10.1186/s13046-025-03504-6)
Supplement: Supplementary file 1 — Supplementary Material 1 [file 13046_2025_3504_MOESM1_ESM.pdf]

Supplementary Materials:

Supplementary Figure 1

A

Target	# binding sites	type	conservation
AMBRA1	1	8-mer	yes
BLOC1S4	1	8-mer	mouse
	2	8-mer	human
ULK1	2	8-mer	no
	2	6-mer	mouse
BNIP3L	1	7-mer-m8	no
CTSB	1	8-mer	human
STX17	1	8-mer	no
	3	7-mer-A1	no
	4	6-mer	human
SNAP29	1	8-mer	no
	1	7-mer-m8	no
	1	7-mer-A1	no
	2	6-mer	human
SCARB2	1	8-mer	no
	1	7-mer-A1	no
	1	6-mer	human
KIF13A	1	8-mer	yes
	1	6-mer	mouse

B

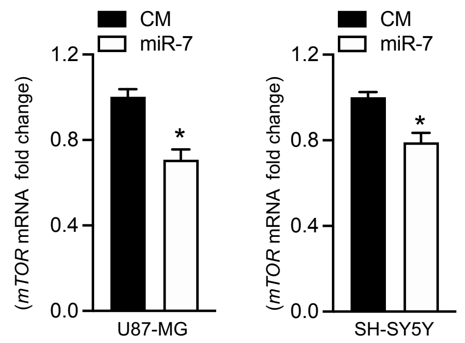

C

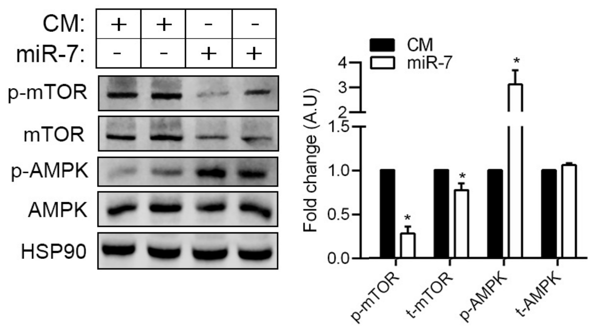

Supplementary Figure 1. MiR-7 targets and regulates the expression of multiple genes related to lysosomal biology, autophagy, mitophagy and the autophagy signaling cascade. A. Table of miR-7 target genes, including the number of 3'UTR binding sites for miR-7, the binding sequence type, and its conservation status in mice and humans. **B.** Representative quantitative PCR *mTOR*, in the indicated cells transfected with control mimics (CM) or miR-7-5p mimics (miR-7). Data represent means ± SEM of three experiments performed in triplicate. **P* <0.05, significantly different from cells transfected with CM (normalized to 1). **C.** Representative Western blot and quantification of phosphorylated and total mTOR and AMPK proteins in mouse neuronal cells (N2a) transfected with CM or miR-7. HSP90 was used as a housekeeping protein. Right graph shows relative protein expression normalized to HSP90. Data correspond to the means ± SEM of three experiments performed in triplicate. **P* <0.05, significantly different from cells transfected with CM (normalized to 1).

Supplementary Figure 2

A

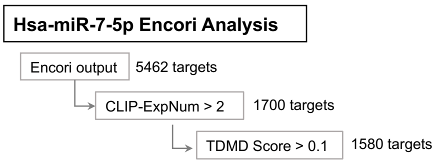

Gene name	ClipExpNum	RBP	PITA	RNA22	miRmap	microT	miRanda	PicTar	TargetScan	TDMDScore	phyloP	PancancerNum
BLOC1S4	4	AGO1-4,AGO2	1	0	1	1	1	0	1	1,0795	0,607	3
BNIP3L	5	AGO1,AGO1-4,AGO2	1	0	0	0	1	0	0	1,1537	0,864	10
CTSB	9	AGO1,AGO1-4,AGO2	1	0	0	1	1	0	1	1,2683	0,893	10
STX17	2	AGO2	1	0	1	1	0	0	0	1,2905	0,038	1
SNAP29	2	AGO2	1	0	1	0	0	0	0	0,3966	-0,612	7
SCARB2	9	AGO1,AGO2	0	0	0	1	0	0	0	0,9374	2,256	9
DHCR24	2	AGO1,AGO1-4	1	0	1	0	0	0	0	0,1177	-0,393	7

B

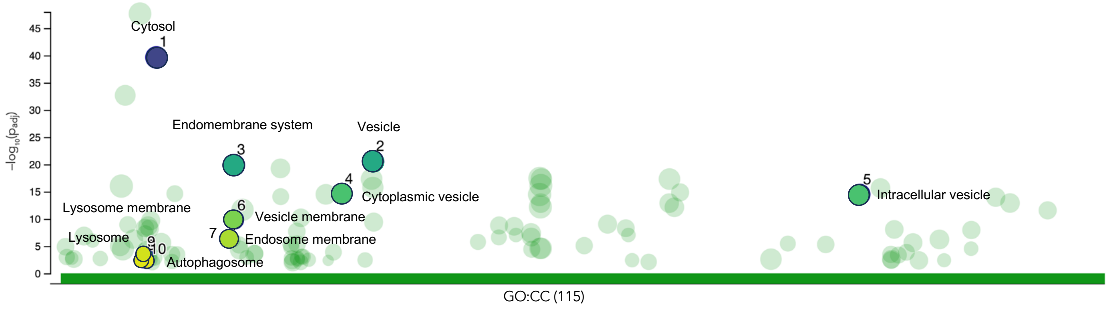

ID	GO Term ID	Term name	-Log10 (P-adj)
1	GO:005829	Cytosol	2.280×10^{40}
2	GO:0031982	Vesicle	2.671×10^{21}
3	GO:0012505	Endomembrane system	1.304×10^{20}
4	GO:0031410	Cytoplasmic vesicle	2.319×10^{15}
5	GO:0097708	Intracellular vesicle	3.075×10^{15}
6	GO:0012506	Vesicle membrane	1.435×10^{10}
7	GO:0010008	Endosome membrane	4.437×10^{7}
8	GO:0005764	Lysosome	2.276×10^{3}
9	GO:0005765	Lysosomal membrane	4.403×104
10	GO:0005776	Autophagosome	5.774×10^{3}

Supplemental Figure 2. Integration of experimental datasets and cross-validation of miR-7 targets involved in autophagy. A. EncoRi analysis pipeline (upper panel) used to identify miR-7 targets with experimental support from CLIP-seq data. The lower table summarizes the main results, displaying selected candidate targets with strong experimental evidence related to autophagy pathways. It includes the number of CLIP experiments, RNA-binding proteins (RBPs), and shows comparative analysis of prediction from multiple algorithms including PITA, RNA22, microT, miRanda, PicTar, and TargetScan. DHCR24 was included as a positive control, as a previously validated target. TDMD score (reflects target-directed miRNA degradation potential), Phylo conservation scores (indicates evolutionary conservation), and PanCancer numbers (shows how many cancers types the miRNA-target interaction). **B**. Gene Ontology (GO) enrichment analysis of the shortlisted miR-7 targets using g:Profiler, confirming significant enrichment of cellular components (CC) associated with vesicles, lysosomal membranes, lysosomes and autophagosomes (highlighted circles). The lower panel lists the previously highlighted CC, including details on the associated GO terms and adjusted *P*-values.

Supplementary Figure 3

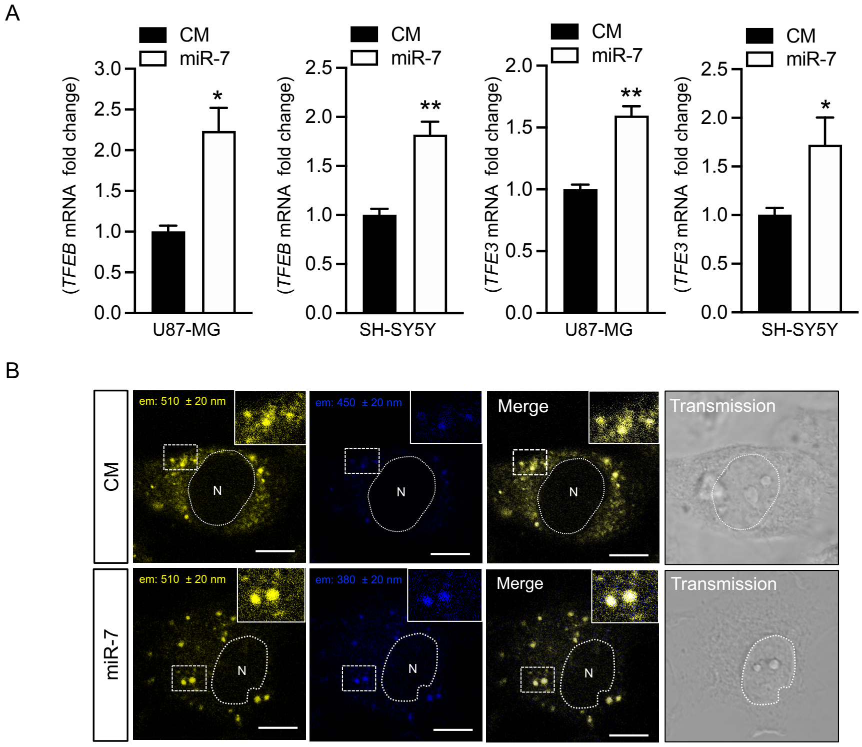

Supplementary Figure 3. MiR-7 interferes with lysosome biogenesis and impairs lysosomal pH. A. RT-qPCR analysis of TFEB and TFE3 mRNA expression in U87-MG cells transfected with control mimic (CM) or miR-7-5p (miR-7). Data correspond to the means ± SEM of three experiments performed in triplicate. **P* <0.05, ***P* <0.01, significantly different from cells transfected with CM (normalized to 1). **B.** Representative confocal images of U87-MG cells treated with LysoSensor Yellow/Blue DND-160 probe in cells transfected with CM or miR-7. Yellow fluorescence represents more acidic lysosomal environment, and blue fluorescence represents more neutral lysosomal environment. N (nuclei). The gray field represents the transmisión mode. Upper insets show 4X magnification. Scale bar: 5 μm.

Supplementary Figure 4

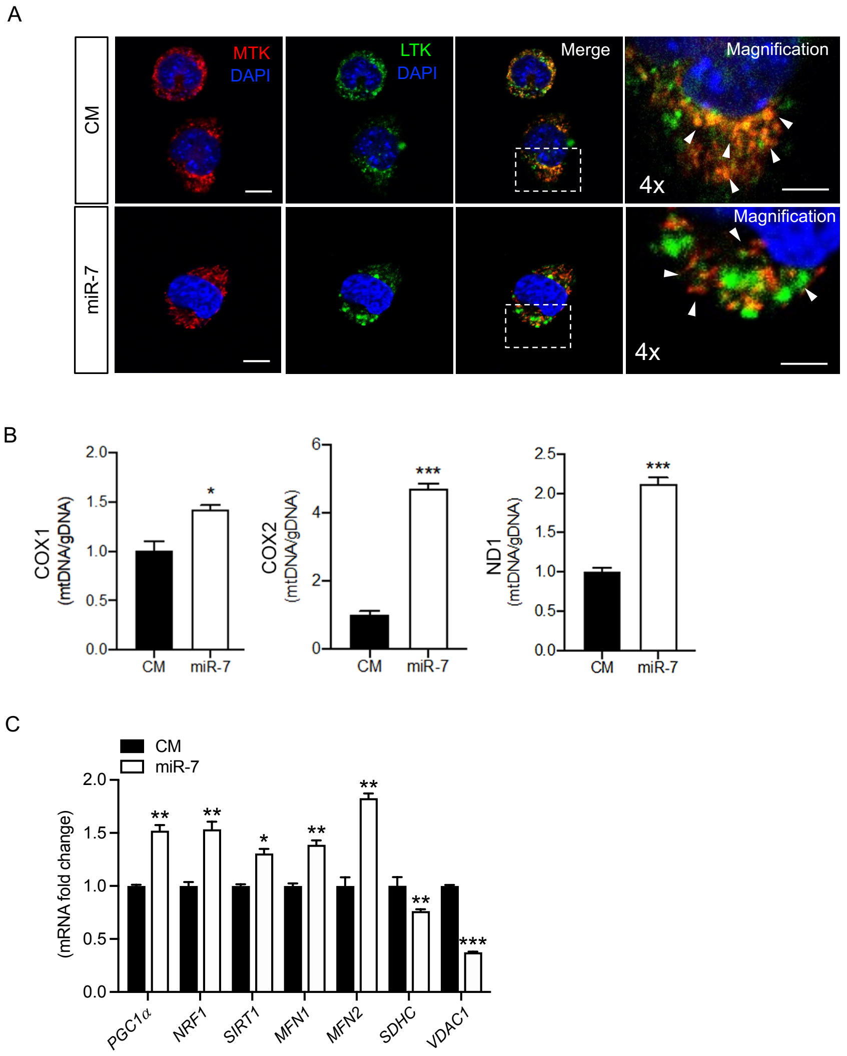

Supplementary Figure 4. MiR-7 regulates mitophagy, increases mtDNA, and modulates key regulatory mitochondrial genes. A. Confocal imaging of Mitotracker (MTK) Red and Lysotracker (LTK) Green staining in CM and miR-7 overexpression conditions. Dapi (blue) stains the nucleus. Right panels show 4X magnification of the insets. Scale bar: 5 µm. **B.** RT-qPCR analysis of mitochondrial DNA (mtDNA) transcripts *COX1*, *COX2* and *ND1* in cells transfected with CM or miR-7. Data represent means ± SEM of three experiments performed in triplicate. **P* <0.05, ****P* <0.001, significantly different from cells transfected with CM (normalized to 1). **C.** RT-qPCR analysis of key genes involved in mitochondrial regulation including *PGC1α*, *NRF1*, *SIRT1*, *MFN1*, *MFN2*, *SDHC* and *VDAC1* in cells transfected with CM or miR-7. Data represent means ± SEM of three experiments performed in triplicate. **P* <0.05, ****P* <0.001, significantly different from cells transfected with CM (normalized to 1).

Supplementary Figure 5

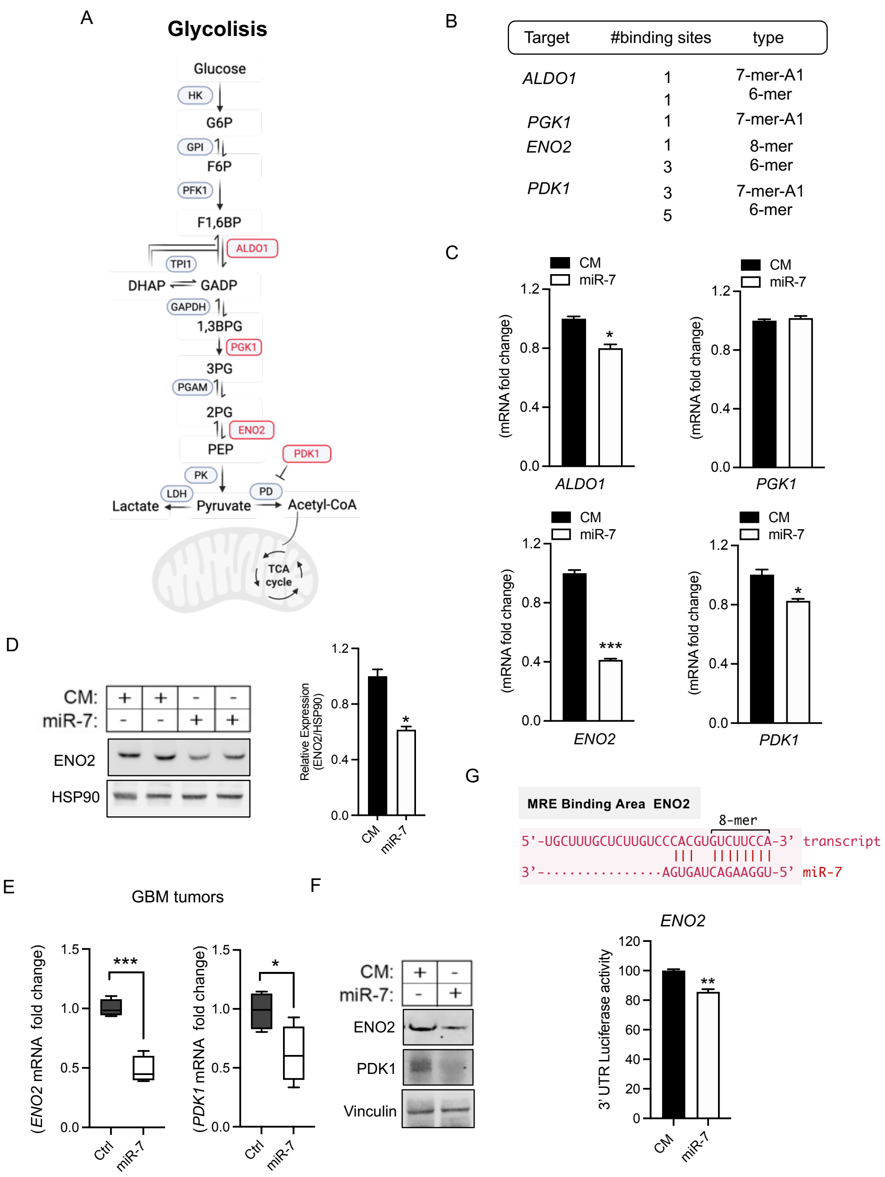

Supplementary Figure 5. Regulation of glycolysis by miR-7 via direct modulation of glycolytic enzymes. A. Diagram of the glycolytic pathway with enzymes predicted to be miR-7 targets shown in red. **B.** Table of miR-7 target genes, including the number binding sites for miR-7 and the binding sequence type in their 3'UTRs. **C.** RT-qPCR analysis *ALDO1*, *PGK1*, *ENO2* in U87-MG cells transfected with CM or miR-7. Data represent means ± SEM of three experiments performed in triplicate. **P* <0.05, ****P* <0.001, significantly different from cells transfected with CM (normalized to 1). **D.** Representative Western Blot of ENO2 in U87-MG cells treated as in C. HSP90 was used as a housekeeping protein. Right graph shows relative protein expression normalized to HSP90. Data correspond to the means ± SEM of three experiments performed in triplicate. **P* <0.05, significantly different from cells transfected with CM (normalized to 1). **E.** RT-qPCR analysis *ENO2 and PDK1* in control (Ctrl) and miR-7 tumors. Data represent means ± SEM (n=4), **P* <0.05, ****P* <0.001, significantly different from Ctrl tumors (normalized to 1). **F.** Representative Western Blot of ENO2 in Ctrl and miR-7 tumors. Vinculin was used as a housekeeping protein. **G.** Luciferase reporter activity of ENO2 3'UTR in HEK293 cells transfected with control mimic (CM) or miR-7-5p (miR-7) and with 3'UTRs wild-type (WT) constructs. Data are expressed as relative luciferase activity compared with control samples cotransfected with CM and represent means ± SEM of three experiments performed in triplicate. **P* < 0.05, ****P* < 0.001, significantly different from cells cotransfected with CM and the WT vectors. Upper panel shows the MRE (miRNA Response Element) binding area within the ENO2 3′UTR, highlighting the predicted 8-mer binding site for miR-7.

Supplementary Figure 6

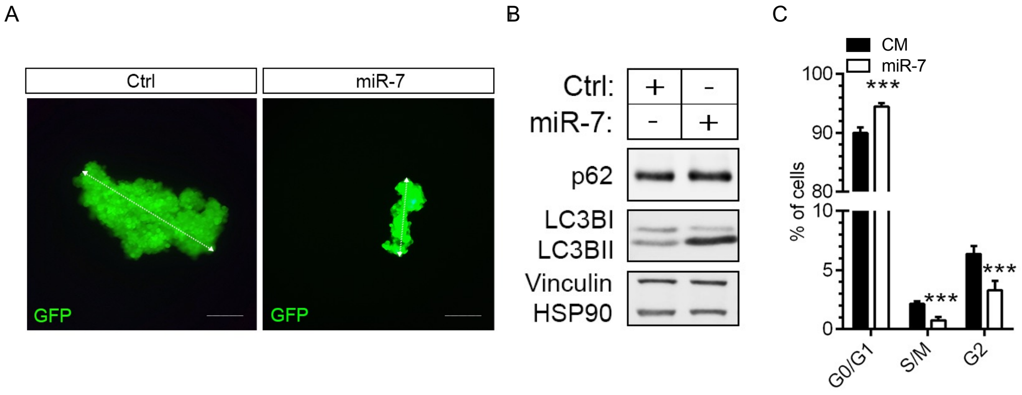

Supplementary Figure 6. MiR-7 disrupts cell growth in a 3D tumorsphere model. A. Fluorescence microscope imaging of control (Ctrl) and U87-miR-7 cell line-derived tumorspheres treated with doxycycline (Dox). Scale bars: 500 nm. **B.** Representative Western blot that shows autophagy markers p62 and LC3BI/II in tumorspheres derived from Ctrl and miR-7 cell lines following Dox treatment. Vinculin and HSP90 were used as loading controls. **C.** Representative analysis of cell cycle in Ctrl and miR-7 cell line-derived tumorspheres after Dox treatment by flow cytometry. Data represent means ± SEM of three experiments performed in triplicate. ****P* <0.001, significantly different from control conditions.

Supplementary Figure 7

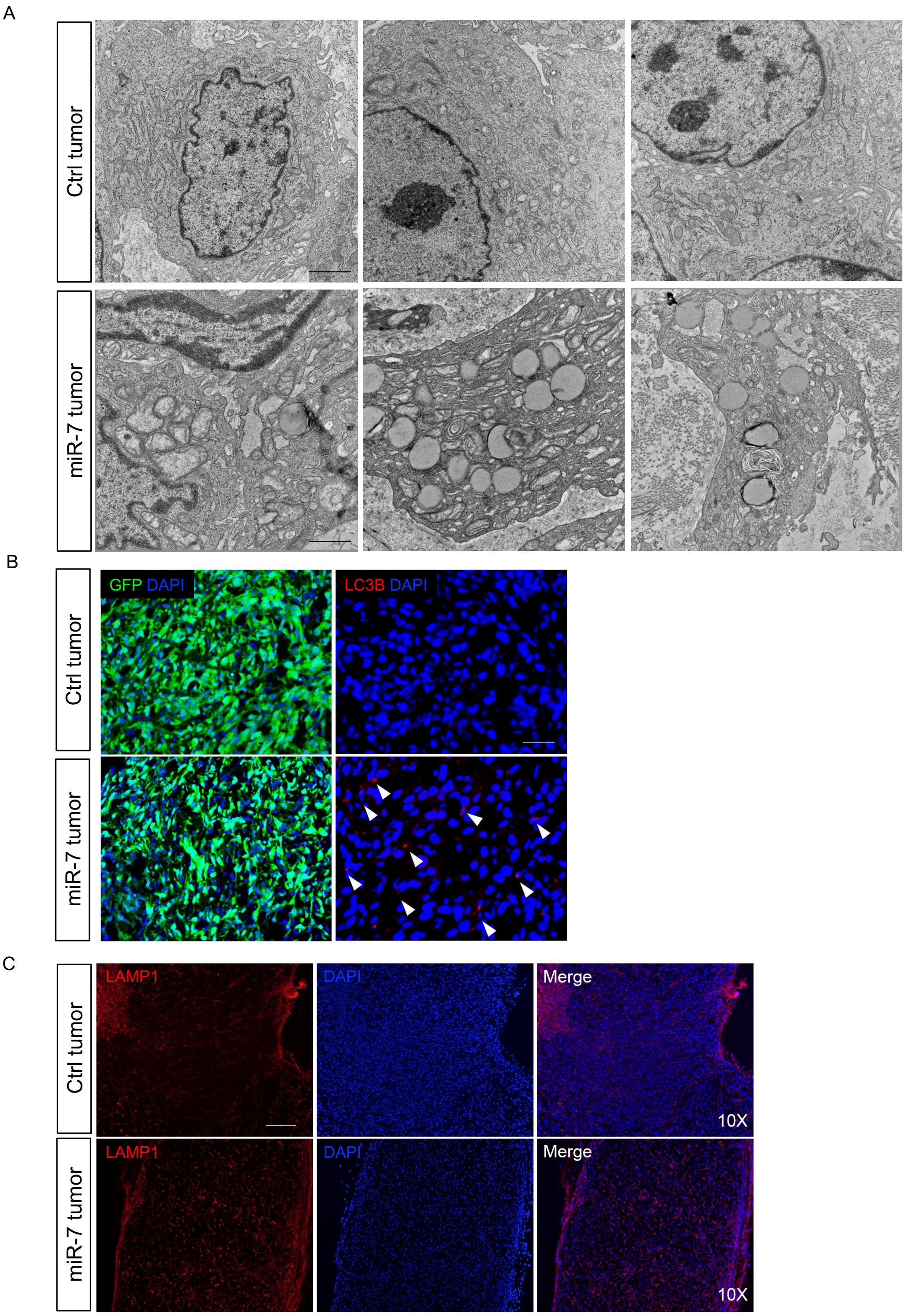

Supplementary Figure 7. MiR-7 promotes accumulation of autophagic vesicles in GBM xenograft tumors. A. Electron microscopy images detailing the accumulation of vesicles and amorphous mitochondria in control (Ctrl) and miR-7 tumors. Scale bar: 1 µm. **B**. Confocal immunofluorescence imaging of Ctrl and miR-7 tumors stained with LC3B (Red). Green indicates GFP expression in the tumor cells and white arrows highlight vesicles. Dapi (blue) stains the nuclei**.** Scale bar: 15 µm. **C.** Representative confocal microscopy images of Ctrl and miR-7 tumors stained with LAMP1 antibody (Red). Dapi (blue) stains the nuclei. Scale bar: 10 µm.

Supplementary Figure 8

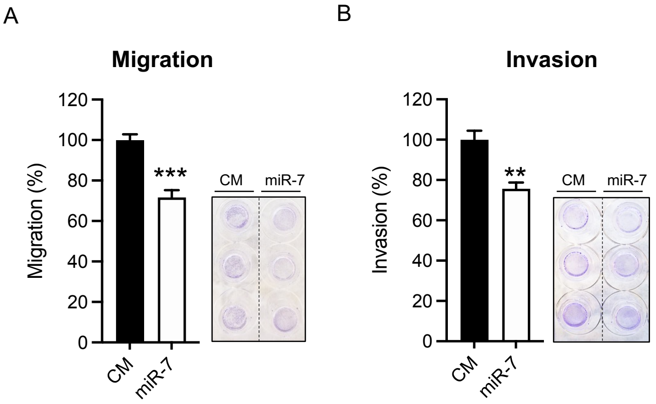

Supplementary Figure 8. MiR-7 attenuates oncogenic parameters in GBM cells. A. Migration rate in U87-MG cells transfected with control mimic (CM) or miR-7-5p (miR-7). **B**. Invasion rate in U87-MG cells treated as in A. Data correspond to the means ± SEM of three experiments performed in triplicate. **P <0.01, ***P <0.001, significantly different from cells transfected with CM (normalized to 100).

Supplementary Figure 9

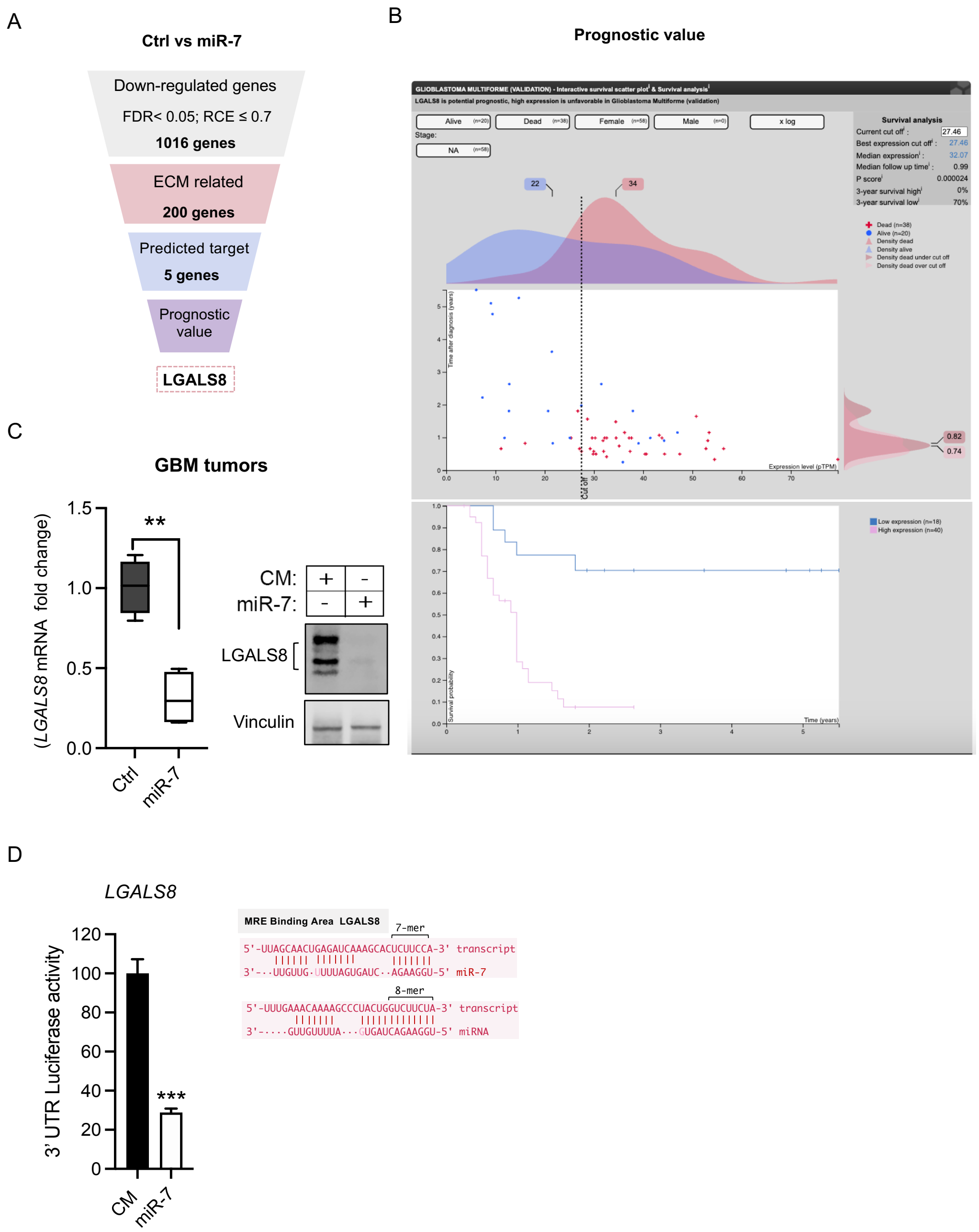

Supplemental Figure 9. Sequential identification and validation of LGALS8 as a direct miR-7 target with prognostic relevance in GBM. A. Schematic overview of the sequential selection strategy applied to transcriptomic data from GBM tumors: significantly downregulated genes in miR-7 tumors were prioritized based on (i) confirmed extracellular matrix (ECM)–related functions, (ii) prognostic significance and correlation with tumor size reduction, and (iii) predicted miR-7 binding sites in the 3′UTR. This approach identified LGALS8 as a prominent ECM-associated candidate gene. **B**. Prognostic analysis of LGALS8 expression obtained from The Human Protein Atlas (www.proteinatlas.org), showing that higher LGALS8 levels are associated with decreased overall survival in GBM patients. **C**. In vivo expression of LGALS8 in xenograft tumors. (Left) RT-qPCR analysis of *LGALS8* mRNA expression in control (Ctrl) and miR-7 tumors. Data represent means ± SEM (n=4), **P* <0.05, ****P* <0.001, significantly different from Ctrl tumors (normalized to 1); (Right) Representative Western blot of LGALS8 protein levels in Ctrl and miR-7 tumors. Vinculin was used as a housekeeping protein. **D.** Luciferase reporter activity of the LGALS8 3′UTR in HEK293 cells transfected with control mimic (CM) or miR-7-5p (miR-7) together with construct containing the wild-type (WT) 3′UTR sequence. Data are expressed as relative luciferase activity compared with control samples cotransfected with CM and represent means ± SEM of three experiments performed in triplicate. **P* < 0.05, ****P* < 0.001, significantly different from cells cotransfected with CM and the WT vectors. Right panel shows the MRE (miRNA Response Element) binding area within the LGALS8 3′UTR, highlighting the predicted 7-mer and 8-mer binding sites for miR-7.
